# Robust delivery of RIG‐I agonists using extracellular vesicles for anti‐cancer immunotherapy

**DOI:** 10.1002/jev2.12187

**Published:** 2022-04-16

**Authors:** Boya Peng, Trinh Mai Nguyen, Migara Kavishka Jayasinghe, Chang Gao, Thach Tuan Pham, Luyen Tien Vu, Eric Yew Meng Yeo, Gracemary Yap, Lingzhi Wang, Boon Cher Goh, Wai Leong Tam, Dahai Luo, Minh TN Le

**Affiliations:** ^1^ Department of Pharmacology and Institute for Digital Medicine Yong Loo Lin School of Medicine National University of Singapore Singapore; ^2^ Department of Surgery Immunology Program Cancer Program and Nanomedicine Translational Program Yong Loo Lin School of Medicine National University of Singapore Singapore; ^3^ Lee Kong Chian School of Medicine Nanyang Technological University Singapore; ^4^ NTU Institute of Structural Biology Nanyang Technological University Singapore; ^5^ Cancer Science Institute of Singapore National University of Singapore Singapore; ^6^ Genome Institute of Singapore, A*STAR Singapore

**Keywords:** cancer, extracellular vesicles, immunotherapy, RIG‐I, RNA delivery

## Abstract

The RIG‐I pathway can be activated by RNA containing 5′ triphosphate, leading to type I interferon release and immune activation. Hence, RIG‐I agonists have been used to induce immune responses against cancer as potential immunotherapy. However, delivery of 5′ triphosphorylated RNA molecules as RIG‐I agonists to tumour cells in vivo is challenging due to the susceptibility of these molecules to degradation. In this study, we demonstrate the use of extracellular vesicles (EVs) from red blood cells (RBCs), which are highly amenable for RNA loading and taken up robustly by cancer cells, for RIG‐I agonist delivery. We evaluate the anti‐cancer activity of two novel RIG‐I agonists, the immunomodulatory RNA (immRNA) with a unique secondary structure for efficient RIG‐I activation, and a 5′ triphosphorylated antisense oligonucleotide with dual function of RIG‐I activation and miR‐125b inhibition (3p‐125b‐ASO). We find that RBCEV‐delivered immRNA and 3p‐125b‐ASO trigger the RIG‐I pathway, and induce cell death in both mouse and human breast cancer cells. Furthermore, we observe a significant suppression of tumour growth coupled with increased immune cell infiltration mediated by the activation of RIG‐I cascade after multiple intratumoral injections of RBCEVs loaded with immRNA or 3p‐125b‐ASO. Targeted delivery of immRNA using RBCEVs with EGFR‐binding nanobody administrated via intrapulmonary delivery facilitates the accumulation of RBCEVs in metastatic cancer cells, leading to potent tumour‐specific CD8^+^ T cells immune response. This contributes to prominent suppression of breast cancer metastasis in the lung. Hence, this study provides a new strategy for efficient RIG‐I agonist delivery using RBCEVs for immunotherapy against cancer and cancer metastasis.

## 1 INTRODUCTION

In recent decades, there have been large strides in the development of immunotherapy beginning with the breakthrough approval of interferon‐alpha2 anti‐tumour cytokine by the U.S. Food and Drug Administration (FDA) (Eno, 2017). Since then, immunotherapeutic drugs have expanded to include anti‐tumour cytokines, checkpoint inhibitors, adoptive transfer T‐cell therapy and cancer vaccines (Waldman et al., 2020); and increasing numbers of immunotherapy medicines have entered into clinical trials and practice (Irvine & Dane, 2020). The key concept of immunotherapy acknowledges that tumours are part of the tumour microenvironment which includes host immune cells (Gajewski et al., 2013), and that the immune system can be harnessed to induce anti‐tumour effects (Rameshbabu et al., 2021).

In recent years, activating the innate immune system through the RIG‐I‐like receptor (RLR) pathway for anti‐cancer therapy has been an active field of research. When a pathogen infects the cell, pattern recognition receptors (PRRs)—which includes the Toll‐like receptors (TLRs), NOD‐like receptors (NDLRs) and RLRs—recognize foreign pathogens and activate an anti‐microbial response (Rameshbabu et al., 2021). RIG‐I is a cytosolic RNA sensor that recognizes RNA sequences with a 5′ triphosphate moiety and binds to short double‐stranded RNAs with higher affinity (Kato et al., 2006; Loo & Gale, 2011). Interestingly, high RIG‐I expression level is correlated with prolonged survival in patients with hepatocellular carcinoma (Hou et al., 2014), but is shown to associate with poor clinical outcome for ovarian cancer (Wolf et al., 2020). Although the role of RIG‐I as a prognostic marker varies across cancer types, its activation consistently induces apoptotic tumour cell death and impacts immune response by increasing infiltration of immune cells into the tumour, enhancing their anti‐cancer effects and reducing immunosuppressive activities (Besch et al., 2009; Bourquin et al., 2020; Das et al., 2019; Duewell et al., 2014; Ellemeier et al., 2013; D. Peng et al., 2020).

A series of preclinical evidence concurs that the success of RIG‐I agonists in immunotherapy hinges on the responsiveness of cancer cells and immune cells to type I interferons (IFNs). Intratumoral delivery of RIG‐I agonists induces apoptosis of pancreatic cancer cells in a type I IFN‐dependent manner and enhances effective cross‐presentation of tumour‐associated antigens by dendritic cells to CD8^+^ T cells, therefore leading to tumour regression and prolonged survival of mice bearing pancreatic cancer (Duewell et al., 2014). Systemic treatment of acute myeloid leukaemia (AML) in humanized mouse model with a RIG‐I agonist decreases AML burden, delays AML progression and sensitizes AML cells to checkpoint blockade. The therapeutic efficacy of RIG‐I agonists is highly dependent on adaptive immunity mediated by CD8^+^ and CD4^+^ T cells in response to type I IFN signalling (Ruzicka et al., 2020).

As many therapeutic RNA oligonucleotides including small interference RNAs (siRNAs) and antisense oligonucleotides, are being developed to target many disease genes that were once considered undruggable (Chery, 2016; Dammes & Peer, 2020; Sehgal et al., 2013), combining gene knockdown with RIG‐I activation is an attractive approach for anti‐cancer therapy. Two research groups have developed bi‐functional siRNAs, which target *BCL‐2* and *TGF‐β*, with 5′ triphosphate modification to simultaneously activate RIG‐I (Ellemeier et al., 2013; Poeck et al., 2008). Both bi‐functional siRNAs were reported to induce anti‐tumour effects.

Despite their great potential, the clinical application of RNA therapeutics continues to be restrained by current inefficient delivery of these molecules to target cells (Dammes & Peer, 2020). One of the most widely used delivery vehicles of RIG‐I agonists is the polymer in vivo‐jetPEI (Duewell et al., 2014; Ellermeier et al., 2013; Heidegger et al., 2019; Poeck et al., 2008; Ruzicka et al., 2020). Whilst it has been reported to be efficient, there have been contrasting reports on its safety. In vivo‐jetPEI tends to induce liver damage and is associated with poor survival rates of mice, an effect attributed to its toxicity (Akita et al., 2011; Hayashi et al., 2012). Thus, developing an effective and safe delivery method is crucial for RNA therapy.

We have previously reported the advantages of using extracellular vesicles (EVs) derived from red blood cells (RBCs) as therapeutic delivery vehicles as it is economical, readily available, easily scalable, non‐immunogenic and non‐oncogenic (Usman et al., 2018). We have demonstrated efficient knockdown of miR‐125b and inhibition of tumour growth using RBCEV‐delivered ASOs in human breast cancer and AML xenograft mouse models via intratumoral and systemic administration, respectively (Usman et al., 2018). Furthermore, there was no observable toxicity associated with RBCEV treatment (Usman et al., 2018). We have also described an EV surface functionalization approach that facilitates EGFR‐targeted delivery of paclitaxel‐loaded RBCEVs to EGFR‐positive lung cancer cells and increases treatment efficacy in vivo (Pham et al., 2021).

Thus, in this study, we sought to evaluate the effectiveness of RBCEVs in delivering RIG‐I agonists, including an immunomodulatory RNA (immRNA) and a bi‐functional ASO for anti‐cancer therapy. The immRNA is a small, stable and highly potent RIG‐I agonist capable of activating RIG‐I and subsequent anti‐viral responses in macrophages and dendritic cells (Ho et al., 2019; Kohlway et al., 2013; Luo et al., 2012; Yong et al., 2019). We hypothesize that delivery of either immRNA or anti‐miR‐125b ASO with a 5′ triphosphate modification (3p‐125b‐ASO) will induce a robust anti‐cancer response. Here, we demonstrated that immRNA and 3p‐125b‐ASO in RBCEVs induce RIG‐I pathway activation, type I IFN production, and apoptosis in breast cancer cells and lung cancer cells in vitro. Intratumoral administration of RBCEVs containing immRNA and 3p‐125b‐ASO suppressed tumour growth of mammary breast cancer and triggered high levels of type I IFN in the tumour microenvironment, immune cell infiltration, and profound tumour cell apoptosis in vivo. Furthermore, we demonstrated that the enzymatic conjugation of RBCEVs with EGFR‐targeting nanobodies facilitates specific uptake of RBCEVs by EGFR‐positive breast cancer cells in vitro. Targeted delivery of immRNA‐loaded RBCEVs conferred improved specific anti‐tumour immune response in EGFR‐positive metastatic breast cancer mouse models.

## 2 RESULTS

### 2.1 RBCEVs can be loaded with small RNAs using multiple methods for the delivery of RNAs to cancer cells

**FIGURE 1:**
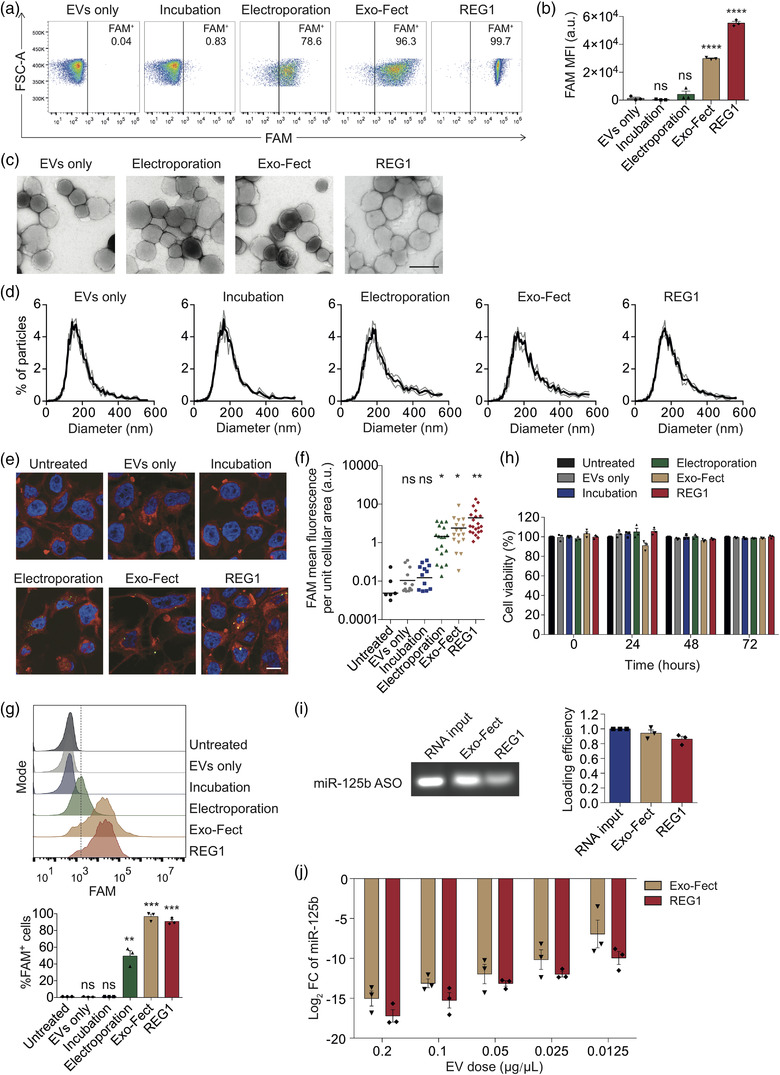
RBCEVs can be loaded with small RNAs using multiple methods for the delivery of RNAs to cancer cells. (a) Flow cytometry analysis of RBCEVs loaded with FAM‐labelled ASO using electroporation, Exo‐Fect and REG1. As a negative control, RBCEVs were incubated with FAM‐ASO without electroporation or loading reagents. (b) Average mean intensity of FAM in RBCEVs loaded with FAM‐labelled ASO using electroporation, Exo‐Fect and REG1 (*n* = 3), compared to the EVs only condition for statistical analysis. (c) Transmission electron microscopy images of FAM‐ASO‐loaded RBCEVs. Scale bar, 200 nm. (d) Size distribution of FAM‐ASO‐loaded RBCEVs, determined using ZetaView^®^ nanoparticle tracking analyzer. (e) Representative immunofluorescent images of CA1a cells taking up FAM‐ASO in RBCEVs. Nuclei were stained with Hoechst (blue). Plasma membranes were stained with CellMask Deep Red (red). Scale bar, 20 μm. (f) Average mean intensity of FAM in CA1a cells as in (e), compared to untreated control for statistical analysis. (g) Flow cytometry analysis of CA1a cells taking up FAM‐ASO‐loaded RBCEVs (*n* = 3), compared to untreated control for statistical analysis. (h) Viability of human breast cancer CA1a cells treated with 0.05 μg/μl NC‐ASO‐loaded RBCEVs at different time points. Viable cells were quantified by normalizing the absorbance readings to the untreated control at each time point (*n* = 3). (i) Loading efficiency of miR‐125b ASO in RBCEVs using Exo‐Fect and REG1, determined using gel electrophoresis (*n* = 3). (j) qPCR analysis of miR‐125b fold change (FC) relative to EVs only condition, normalized to *U6B* in CA1a cells treated with different doses of RBCEVs containing miR‐125b ASO (*n* = 3). All bar graphs represent mean ± SEM. ns, not significant; *p < 0.05, **p < 0.01, ***p < 0.001, and ****p < 0.0001 determined by Student's two‐tailed *t*‐test

RBCEVs were purified according to our previous protocol (Usman et al., 2018). Purified RBCEVs were enriched in common EV markers, such as ALIX and TSG101, as well as haemoglobin A (HBA), the major RBC protein (Figure S1A). We also found that RBCEVs express glycophorin A (GPA) on the surface as an exclusive marker for EVs originated from human RBCs (Figure S1A‐B). The cytoskeleton protein β‐actin was largely absent in RBCEVs, suggesting that purified RBCEVs did not contain cellular debris (Figure S1A). Additionally, purified RBCEVs were homogenous in size, 120–200 nm in diameter, as determined using a nanoparticle analyzer (Figure 1d).

In order to deliver therapeutic RNAs to cancer cells, we loaded RBCEVs with small RNAs using multiple methods including electroporation, Exo‐Fect‐mediated transfection and REG1‐mediated transfection. RBCEVs were separated from RNA‐transfectant complexes using three rounds of centrifugation. Bead‐assisted flow cytometry analysis of RBCEVs loaded with FAM‐ASO revealed that FAM‐ASO was loaded efficiently into RBCEVs using three different methods (Figure 1a‐b). The loading efficiency by Exo‐Fect and REG1 were relatively higher than that by electroporation (Figure 1a‐b). We also observed that the loading with REG1 yielded a homogenous population of RBCEVs with strong FAM fluorescence (Figure 1a). By contrast, a simple incubation did not facilitate the entry of FAM‐ASO into RBCEVs (Figure 1a‐b). Transmission electron microscopy revealed that following REG1 transfection, RBCEVs retained a similar morphology to untreated control, while the electroporated and Exo‐fect‐transfected RBCEVs showed signs of aggregation as indicated by the greater extent of ruffled membranes (Figure 1c). Nanoparticle tracking analysis also revealed that REG1 maintained the size of RBCEVs (Figure 1d). Upon electroporation and Exo‐Fect transfection, RBCEVs were prone to aggregation as evidenced by the increase in the proportion of EVs with larger particle size, ∼400–600 nm in diameter (Figure 1d).

We further investigated the uptake of RBCEVs loaded with FAM‐ASO using three loading methods by human breast cancer MCF10CA1a (CA1a) cells. Flow cytometry and immunofluorescent analysis demonstrated that CA1a cells readily took up RBCEVs containing FAM‐ASO (Figure 1e‐g). Importantly, confocal imaging revealed that the FAM signal was present inside the cells (Figure 1e). Exo‐Fect‐transfected RBCEVs exhibited the highest uptake by CA1a cells shown by flow cytometry (Figure 1g), whereas REG1‐transfected RBCEVs were taken up the most by CA1a cells shown by immunofluorescent analysis (Figure 1e‐f). To evaluate the potential toxic effects of three loading methods, CA1a cells were incubated with electroporated, Exo‐Fect‐transfected and REG1‐transfected RBCEVs for three days. CCK‐8 assay analysis showed that electroporation and REG1 were non‐toxic to CA1a cells, whereas Exo‐fect exhibited a mild toxicity to the cells after 24‐h incubation but not at later time points (Figure 1h). Overall, RBCEVs treated with REG1 and Exo‐Fect were comparable in delivery. Both REG1 and Exo‐Fect performed better than electroporation for the loading of RNA into RBCEVs. As such, we used Exo‐Fect and REG1 as transfectants for RBCEVs in the subsequent experiments. To estimate the loading efficiency of the transfectants, RBCEVs were transfected with miR‐125b ASOs using Exo‐Fect and REG1, and then lysed by detergents. miR‐125b ASOs were separated by gel electrophoresis (Figure 1i). Based on the band fluorescent intensity, we found approximately ∼94.2% and ∼86% of 125b‐ASO loaded in RBCEVs by Exo‐Fect and REG1, respectively (Figure 1i). The two transfection methods conferred strong inhibition of miR‐125b in CA1a cells with similar efficiency as shown by a dose‐response assay (Figure 1j).

### 2.2 RBCEVs deliver immunomodulatory RNA to activate the RIG‐I pathway and induce immunogenic cell death of cancer cells

**FIGURE 2:**
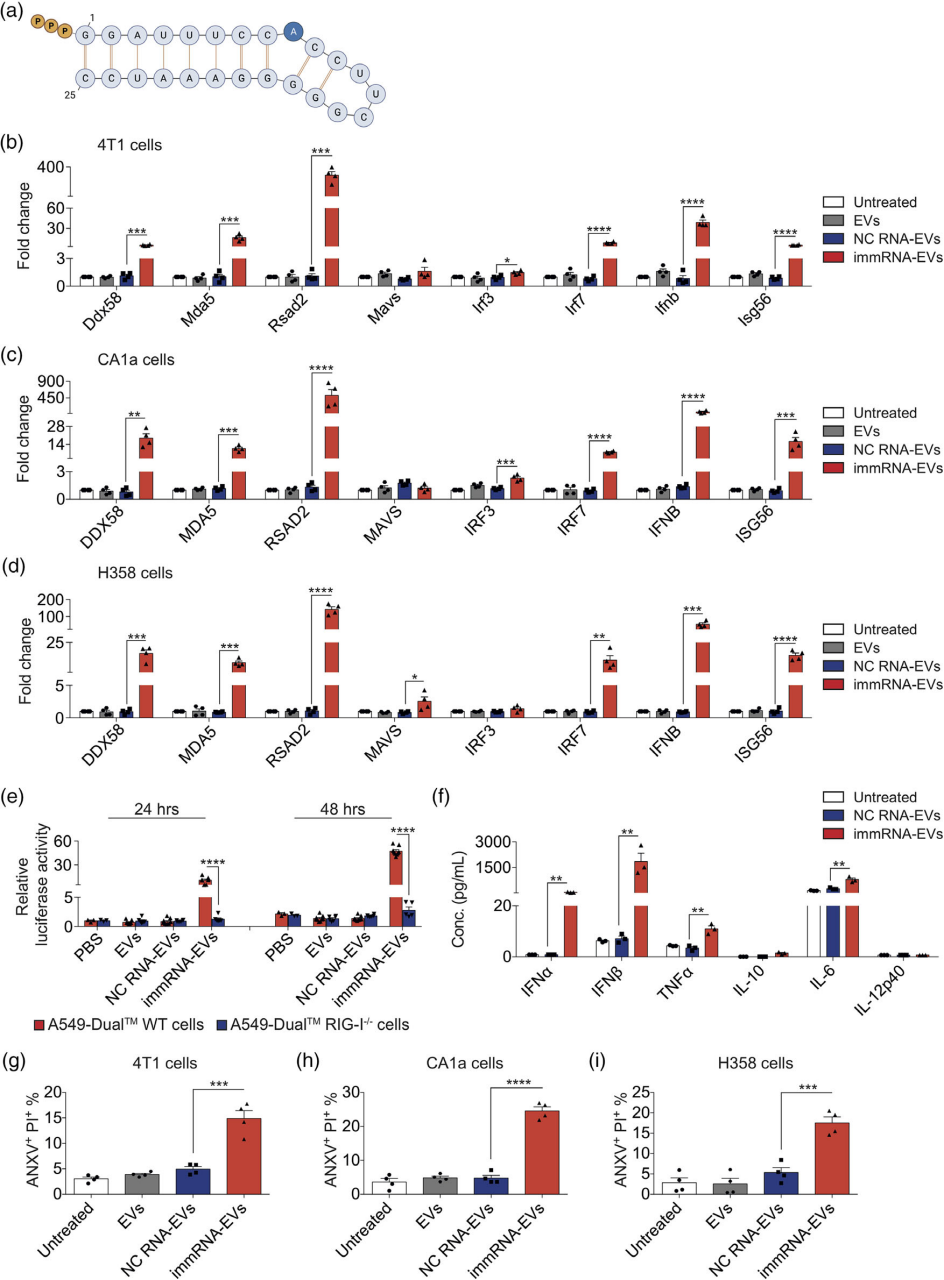
RBCEVs deliver immunomodulatory RNA to activate the RIG‐I pathway and induce immunogenic cell death in cancer cells. (a) The design of immRNA with 5′ triphosphate (ppp). (b–d) qPCR analysis of RIG‐I encoding mRNA (*Ddx58*) and its downstream effectors relative to *Gapdh* in mouse breast cancer 4T1 cells (b), human breast cancer CA1a cells (c) and human lung cancer H358 cells (d) treated with 0.1 μg/μl unloaded RBCEVs, NC‐RNA‐loaded RBCEVs, and immRNA‐loaded RBCEVs for 24 h (*n* = 4, RNA loaded using REG1). (e) Average luciferase activity in A549‐Dual™ and A549‐Dual™ RIG‐I^−/−^ cells treated with PBS, 0.05 μg/μl unloaded RBCEVs, NC‐RNA‐loaded RBCEVs and immRNA‐loaded RBCEVs for 24 and 48 h (*n* = 5–9). (f) Multiplex immunoassay analysis of cytokines in the conditioned media of 4T1 cells treated with 0.1 μg/μl unloaded RBCEVs, NC‐RNA‐loaded RBCEVs and immRNA‐loaded RBCEVs for 48 h (*n* = 3). (g–i) Flow cytometry analysis showing the average percentage of ANXV^+^PI^+^ population in 4T1 cells (g), CA1a cells (h) and H358 cells (i) after a treatment with 0.1 μg/μl unloaded RBCEVs, NC‐RNA‐loaded RBCEVs and immRNA‐loaded RBCEVs for 72 h (*n* = 4). All bar graphs represent mean ± SEM. *p < 0.05, **p < 0.01, ***p < 0.001, and ****p < 0.0001 determined by Student's two‐tailed *t*‐test

The Luo group previously developed a series of immRNA species as potent RIG‐I agonists in human skin dendritic cells and macrophages (Ho et al., 2019; Yong et al., 2019). Among the immRNA variants, 3p10LA9 showed the strongest cellular IFNs‐producing activity. To examine the effects of the immRNA 3p10LA9 (Figure 2a) on breast cancer cells, we transfected RBCEVs with immRNA using REG1 and incubated the immRNA‐loaded RBCEVs with the highly metastatic mouse breast cancer 4T1 cells for 24 h. A scrambled RNA was used as a negative control (NC). qPCR analysis revealed that the uptake of immRNA‐loaded RBCEVs by 4T1 cells led to significant up‐regulation of cellular RLRs, *Ddx58* (the gene encoding RIG‐I) and *Mda5*, as well as RLR downstream effectors including *Irf3*, *Irf7*, *Ifnb*, *Rsad2 (Viperin)* and *Isg56*, as compared to NC‐RNA‐loaded RBCEVs (Figure 2b). The effects of immRNA were also assessed in human triple‐negative basal‐like breast cancer (TNBC) CA1a cells, which is a progressively aggressive and metastatic derivative of MCF10A cells, with the same doses of immRNA‐loaded RBCEVs, yielding a similar up‐regulation of RIG‐I related genes. The uptake of immRNA‐loaded RBCEVs by CA1a cells up‐regulated the cellular levels of *DDX58*, *MDA5*, *IRF3*, *IRF7*, *IFNB*, *RSAD2*, and *ISG56* significantly (Figure 2c). Similarly, human TNBC MDA‐MB‐468 cells were highly responsive to immRNA‐loaded RBCEVs as shown by substantial up‐regulation of *DDX58*, *MDA5*, *IRF7*, *IFNB*, *RSAD2*, and *ISG56* (Figure S2A). However, immRNA‐loaded RBCEVs did not induce RIG‐I activation in human TNBC MDA‐MB‐231 cells, which have very low RIG‐I expression according to Biswas et al. (2012) and our own data (Figure S2B).

We also tested the delivery of immRNA to lung cancer cells. Human lung cancer NCI‐H358 (H358) cells showed significant up‐regulation of *DDX58*, *MDA5*, *MAVS*, *IRF7*, *IFNB, RSAD2*, and *ISG56*, after incubation with immRNA‐loaded RBCEVs (Figure 2d). Remarkably, *RSAD2*, known as an IFN‐stimulated gene, increased by 305‐fold, 518‐fold, 265‐fold and 143‐fold, respectively, in 4T1, CA1a, MDA‐MB‐468, and H358 cells (Figures 2b–d and S2A).

To assess the effects of immRNA on normal cells, untransformed mammary gland epithelial MCF10A cells were incubated with immRNA‐loaded RBCEVs, which led to a slight increase in the level of *DDX58* and *RSAD2* but not other RIG‐I‐related genes in the cells (Figure S2C). We also generated mouse lung epithelial (mLE) cells using a previously established protocol (Kasinski & Slack, 2013). Surface marker EpCAM of the cells was determined by flow cytometry (Figure S1C). The uptake of immRNA‐loaded RBCEVs by lung epithelial cells led to significant up‐regulation of *Ddx58*, *Mda5*, *Irf7*, *Rsad2*, and *Isg56*. *Ifnb* was not detectable in the cells, whereas *Ifna4*, *Ifna11*, and *Ifna12* were up‐regulated significantly (Figure S2D).

We further assessed the type‐I‐IFNs‐producing activity induced by immRNA. We incubated immRNA‐loaded RBCEVs with human lung epithelial carcinoma A549‐Dual™ and A549‐Dual™ RIG‐I^−/−^ cells. These two cell lines secrete the interferon regulatory factor (IRF) pathway mediated Lucia luciferase under the control of an ISG54 minimal promoter in conjunction with five IFN‐stimulated response elements. As a result, the wild‐type (WT) A549‐Dual™ cells incubated with immRNA‐RBCEVs were responsive to this RIG‐I activator as shown by the increased luciferase activity after 24 and 48 h (Figure 2e). Removal of the RIG‐I gene from the cells resulted in complete abrogation of IFN signalling in response to immRNA (Figure 2e), confirming RIG‐I as the primary immRNA sensor in the cells. To evaluate the effect of immRNA on the inflammatory response, we measured the concentrations of key inflammatory mediators in 4T1‐conditioned media. As expected, 4T1 cells treated with immRNA‐loaded RBCEVs secreted substantially higher levels of IFNα (∼55.95 pg/ml) and IFNβ (∼1844.33 pg/ml) (Figure 2f). Furthermore, immRNA‐RBCEVs taken up by 4T1 cells also induced the secretion of proinflammatory cytokines TNFα (∼10.91 pg/ml) and IL‐6 (∼793.22 pg/ml) (Figure 2f).

The induction of immunogenic cell death in cancer cells was subsequently quantified using Annexin V (ANXV) and Propidium Iodide (PI) staining for each EV treatment. After 72 h of incubation, immRNA‐loaded RBCEVs induced apoptosis of 4T1 cells (∼15.9%) (Figures 2g and S2E), CA1a cells (∼24.6%) (Figures 2h and S2E), H358 cells (∼17.6%) (Figures 2i and S2E), and MDA‐MB‐468 cells (∼16.0%) (Figure S2E‐F) as shown by the average percentage of ANXV^+^PI^+^ population. ImmRNA‐loaded RBCEVs failed to induce cell death in RIG‐I‐low MDA‐MB‐231 cells (Figure S2E‐F), indicating that RIG‐I is an essential receptor in response to immRNA. MCF10A cells and mLE cells did not succumb to apoptosis after the incubation with immRNA‐loaded RBCEVs (Figure S2E‐F). These data suggest that immRNA‐loaded RBCEVs activate the cellular RIG‐I pathway, triggering type I IFN secretion and inducing cell death in cancer cells, while concurrently sparing non‐malignant cells.

### 2.3 RBCEVs deliver bi‐functional ASOs to simultaneously inhibit oncogenic miR‐125b and activate the RIG‐I pathway leading to cell death in cancer cells

**FIGURE 3:**
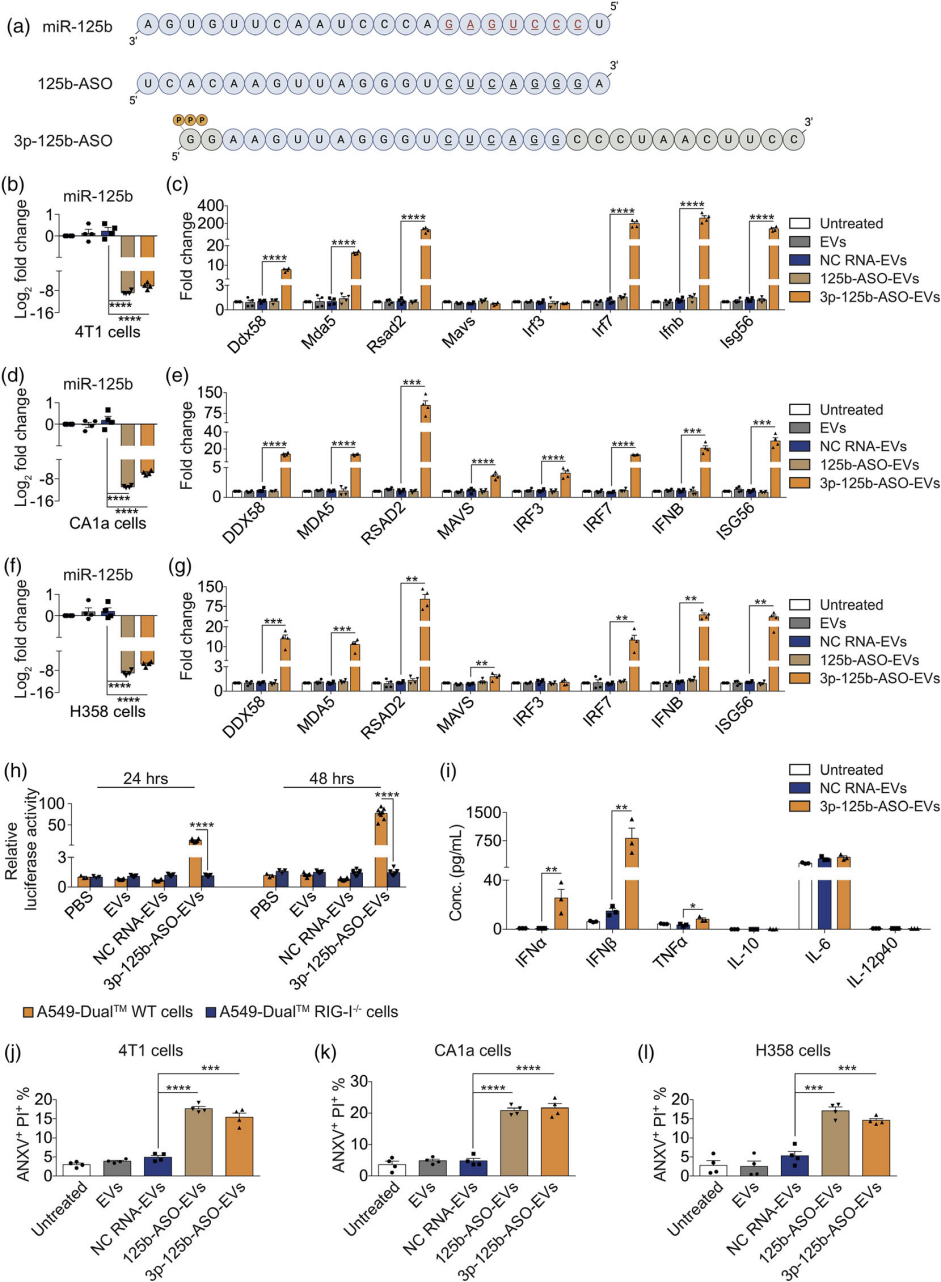
RBCEVs deliver bi‐functional ASOs to simultaneously inhibit oncogenic miR‐125b and activate the RIG‐I pathway leading to cell death in cancer cells. (a) miR‐125b sequence and the design of ASO against miR‐125b with and without 5′ triphosphate. The seed sequence of miR‐125b is colored in red and underlined. A triphosphate group (PPP) was added to the 5′ end of 3p‐125b‐ASO whereas the first four nucleotides were replaced with GG. A short sequence (grey circles) was added to the 3′ end of 3p‐125b‐ASO during IVT. (b) qPCR analysis of miR‐125b relative to *snoRNA234* in 4T1 cells treated with 0.1 μg/μl unloaded or NC‐RNA‐loaded, 125b‐ASO‐loaded and 3p‐125b‐ASO‐loaded RBCEVs for 24 h (*n* = 4, RNA loaded using REG1). (c) qPCR analysis of *Ddx58* and its downstream effectors relative to *Gapdh* in 4T1 cells treated with RBCEVs as in (b). (d) qPCR analysis of miR‐125b relative to *U6B* in CA1a cells treated with RBCEVs as in (b). (e) qPCR analysis of *DDX58* and its downstream effectors relative to *GAPDH* in CA1a cells treated with RBCEVs as in (b). (f) qPCR analysis of miR‐125b relative to *U6B* in H358 cells treated with RBCEVs as in (b). (g) qPCR analysis of *DDX58* and its downstream effectors relative to *GAPDH* in H358 cells treated with RBCEVs as in (b). (h) Average luciferase activity in A549‐Dual™ and A549‐Dual™ RIG‐I^−/−^ cells treated with PBS, 0.05 μg/μl unloaded RBCEVs, NC‐RNA‐loaded RBCEVs and 3p‐125b‐ASO‐loaded RBCEVs for 24 and 48 h (*n* = 5–8). (i) Multiplex immunoassay analysis of cytokines in the conditioned media of 4T1 cells treated with 0.1 μg/μl unloaded, NC‐RNA‐loaded and 3p‐125b‐ASO‐loaded RBCEVs for 48 h (*n* = 3). (j–l) Flow cytometry analysis revealing the average percentage of ANXV^+^PI^+^ population in 4T1 cells (j), CA1a cells (k) and H358 cells (l) after a treatment with 0.1 μg/μl unloaded, NC‐RNA‐loaded, 125b‐ASO‐loaded and 3p‐125b‐ASO‐loaded RBCEVs for 72 h (*n* = 4). All bar graphs represent mean ± SEM. **p < 0.01, ***p < 0.001, and ****p < 0.0001 determined by Student's two‐tailed *t*‐test

After confirming that 5′ triphosphate immRNA could effectively trigger RIG‐I activation, we sought to implement a combinatorial approach that incorporates ASO‐mediated oncogene silencing and RIG‐I‐mediated immune activation simultaneously. Previous studies on miR‐125b uncovered its important role in oncogenesis (B. Peng et al., 2021). It has been reported to promote tumorigenesis and cancer progression in multiple types of cancer including metastatic and drug‐resistant breast cancer. To this end, we designed a miR‐125b ASO coupled with a triphosphate group at the 5′ end (3p‐125b‐ASO) (Figure 3a). To improve the yield of the 3p‐125b‐ASO, the first four nucleotides of the original 125b‐ASO (full‐length complementary to miR‐125b) were removed and replaced with two guanine nucleotides. We also tested a couple of 3′ end truncations. A dsDNA template of the modified 125b‐ASO sequence was inserted after the T7 promoter for efficient in vitro transcription (IVT) according to our established protocol (Luo et al., 2012; Yong et al., 2019). This resulted in a longer product due to the product RNA rebound to the T7 RNA polymerase and self‐primed (in cis) generation of a hairpin duplex (Figure S3A) (Gholamalipour et al., 2018). The sequence of the IVT‐produced 3p‐125b‐ASO was confirmed by sequencing.

To assess the functional impact of 3p‐125b‐ASO in cancer cells, we delivered 3p‐125b‐ASO using RBCEVs. Following incubation of ASO‐loaded RBCEVs with cancer cells for 24 h, we compared the miR‐125b silencing activity of the IVT‐produced 3p‐125b‐ASO with the unmodified miR‐125 ASO (125b‐ASO) carrying a free 5′‐OH group using qPCR. We observed that unmodified 125b‐ASO and modified 3p‐125b‐ASO inhibited endogenous miR‐125b levels to a similar extent in 4T1 cells, CA1a cells and H358 cells as compared to NC‐RNA‐loaded RBCEVs (Figure 3b, d, f). Thus, miR‐125b‐silencing activity was not impeded by the presence of the triphosphate group at the 5′ end and other changes in sequence and structure of the ASO. We next assessed RIG‐I signalling in cancer cells in response to the treatment with 3p‐125b‐ASO‐loaded RBCEVs. The uptake of 3p‐125b‐ASO‐loaded RBCEVs induced significant up‐regulation of *Ddx58*, *Mda5*, *Irf7*, *Ifnb*, *Rsad2*, and *Isg56* in 4T1 cells (Figure 3c); *DDX58*, *MDA5*, *MAVS*, *IRF3*, *IRF7*, *IFNB*, *RSAD2*, and *ISG56* in CA1a cells (Figure 3e); *DDX58*, *MDA5*, *MAVS*, *IRF7*, *IFNB*, *RSAD2*, and *ISG56* in H358 cells, as compared to NC‐RNA‐loaded RBCEVs (Figure 3g). Notably, silencing of miR‐125b by the unmodified 125b‐ASO had no effect on RIG‐I activation in the three cell lines (Figure 3c, e, g). Similar to immRNA‐loaded RBCEVs, 3p‐125b‐ASO‐loaded RBCEVs showed no effect on MDA‐MB‐231 cells, modest effects on MCF10A cells, and up‐regulated *Ifna* but not *Ifnb* via RIG‐I activation in mLE cells (Figure S3B–D).

To validate the activity of type I IFNs secretion induced by 3p‐125b‐ASO, we repeated the reporter assay using A549‐Dual™ and A549‐Dual™ RIG‐I^−/−^ cells. RIG‐I stimulation by 3p‐125b‐ASO‐loaded RBCEVs significantly induced the secretion of IFNs in A549‐Dual™ WT cells after 24 and 48 h in comparison with A549‐Dual™ RIG‐I^−/−^ cells (Figure 3h). RIG‐I knockout confirmed the critical role of RIG‐I for inducing IFN secretion. Likewise, the cytokine immunoassay analysis revealed that 4T1 cells taking up 3p‐125b‐ASO‐loaded RBCEVs released significantly higher levels of IFNα (∼25.42 pg/ml), IFNβ (∼815.41 pg/ml), and TNFα (∼8.23 pg/ml) (Figure 3i).

To examine the functional impact of 3p‐125b‐ASO on intrinsic apoptosis in cancer cells, we incubated cancer cells with 3p‐125b‐ASO‐RBCEVs for 72 h. The treatment with 3p‐125b‐ASO‐RBCEVs strongly induced apoptosis in 4T1 (∼15.5%) (Figures 3j and S3E), CA1a (∼21.7%) (Figures 3k and S3E), and H358 cells (∼14.6%) (Figures 3l and S3E), similar to the effect of the original 125b‐ASO, determined by ANXV and PI binding. 3p‐125b‐ASO‐RBCEVs also induced cell death in MDA‐MB‐231 cells, and exerted modest cytotoxicity to non‐malignant cells, which is an effect attributed to the inhibition of miR‐125b in the cells (Figure S3E‐F). Of note, the combinatorial treatments of immRNA‐RBCEVs and 3p‐125b‐ASO‐RBCEVs in 4T1 cells and CA1a cells did not enhance the effect of each agent on RIG‐I activation and apoptosis (Figure S3G‐H). In contrast, the combination of immRNA‐RBCEVs and 3p‐125b‐ASO‐RBCEVs resulted in less activity of RIG‐I response pathway and exhibited less effect on apoptosis than single‐agent treatments (Figure S3G‐H). Together, these data suggest that the bi‐functional 3p‐125b‐ASO comprises two distinct and independent functions, which can simultaneously inhibit oncogenic miR‐125b and activate RIG‐I signalling, leading to immunogenic death of cancer cells.

### 2.4 Intratumoral delivery of immRNA suppresses breast cancer growth by triggering RIG‐I mediated immune responses

**FIGURE 4:**
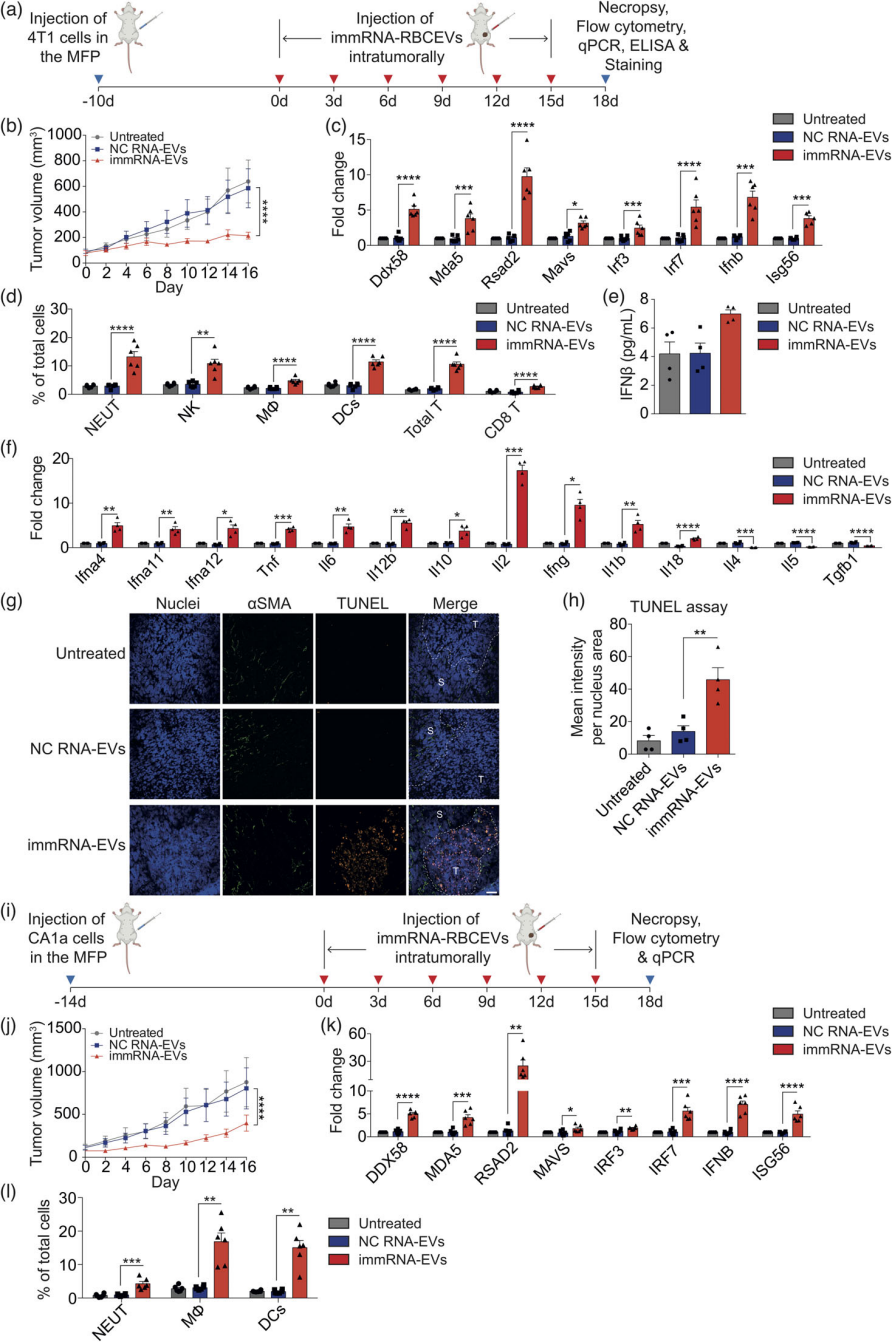
Intratumoral delivery of immRNA suppresses breast cancer growth by triggering RIG‐I mediated immune responses. (a) Schematic treatment of mouse 4T1 mammary tumours in BALB/c mice with intratumoral delivery of immRNA in RBCEVs. (b) Volume of 4T1 tumours injected intratumorally with 2.5 mg/kg RBCEVs containing immRNA or NC RNA every three days (*n* = 6 mice). (c) qPCR analysis of the RIG‐I pathway gene expression relative to *Gapdh* in untreated and treated 4T1 tumours (*n* = 6 mice). (d) Flow cytometry analysis of immune cells in untreated and treated 4T1 tumours (*n* = 6 mice), presented as the average percentage of each subset in total cells. NEUT, neutrophils; NK, natural killer cells; MΦ, macrophages; DCs, dendritic cells. (e) ELISA quantification of IFNβ in the sera of mice with 4T1 tumours (*n* = 4 mice). (f) qPCR analysis of cytokine gene expression relative to *Gapdh* in untreated and treated 4T1 tumours (*n* = 4 mice). (g) Representative images of TUNEL staining (orange fluorescence) of untreated and treated 4T1 tumour sections. Cancer‐associated fibroblasts (green) were stained with anti‐αSMA antibody. Nuclei were stained with Hoechst (blue). Scale bar, 50 μm. T, Tumour; S, Stroma. (h) Average mean intensity per nucleus area of TUNEL staining signals (*n* = 4 mice). (i) Schematic treatment for human CA1a mammary tumours in NSG‐SGM3 mice with intratumoral delivery of immRNA‐loaded RBCEVs. (j) Volume of CA1a tumours injected intratumorally with 2.5 mg/kg RBCEVs loaded with immRNA or NC RNA every three days (*n* = 6 mice). (k) qPCR analysis of RIG‐I pathway gene expression relative to *GAPDH* in untreated and treated CA1a tumours (*n* = 6 mice). (l) Flow cytometry analysis of immune cells in untreated and treated CA1a tumours (*n* = 6 mice). All bar graphs represent mean ± SEM. *p < 0.05, **p < 0.01, ***p < 0.001, and ****p < 0.0001 determined by Student's two‐tailed *t*‐test

To examine the anti‐tumour activity of immRNA in vivo, we implanted 4T1 cells in the fourth mammary fat pad (MFP) of BALB/c mice (Figure 4a). We treated the tumours with RBCEVs containing immRNA or NC RNA intratumorally every three days and monitored tumour growth (Figure 4a). We found that the treatment with immRNA‐loaded RBCEVs significantly dampened 4T1 tumour growth as compared to NC‐RNA‐loaded RBCEVs (Figure 4b). The mice were sacrificed and tumours were excised on day 18 when the untreated tumours reached ∼15 mm in diameter. qPCR analysis of the dissociated tumour cells revealed that the delivery of immRNA‐loaded RBCEVs led to the up‐regulation of *Ddx58, Mda5, Mavs*, *Irf3*, *Irf7*, *Ifnb*, *Rsad2*, and *Isg56* in the tumours (Figure 4c). To confirm the cytotoxic effects of immRNA‐loaded RBCEVs on the tumours, we conducted TUNEL staining, and co‐stained the tumour sections with an antibody for α‐smooth muscle actin (αSMA) to mark cancer‐associated fibroblasts, the most abundant cell type in the tumour stroma. We observed increased apoptosis in tumours treated with immRNA‐loaded RBCEVs, compared to the treatment with NC‐RNA‐loaded RBCEVs (Figure 4g‐h). Furthermore, TUNEL positive cells gathered at the tumour bulk rather than within the stromal compartment (Figure 4g). Interestingly, we found increased infiltration of neutrophils (CD45^+^CD11b^+^Ly6G/C^+^), natural killer (NK) cells (CD45^+^CD11b^+^CD49b^+^), macrophages (CD45^+^CD11b^+^F4/80^+^), dendritic cells (DCs) (CD45^+^CD11c^+^), total T cells (CD45^+^CD3ε^+^), and CD8^+^ T cells (CD45^+^CD3ε^+^CD8^+^) in the tumours treated with immRNA‐RBCEVs in comparison with NC‐RNA‐loaded RBCEVs (Figures 4d and S4A). Additionally, the serum IFNβ level was measured at the endpoint. Intratumoral administration of immRNA‐loaded RBCEVs slightly increased serum IFNβ level (∼6.99 pg/ml) in mice (Figure 4e). To further characterize the cytokine milieu in the tumours, we conducted qPCR of cytokine‐encoding genes in the dissociated tumour cells. The results revealed that immRNA‐loaded RBCEVs significantly increased the mRNA levels of *Ifna4*, *Ifna11*, and *Ifna12*, and multiple pro‐inflammatory cytokine genes, including *Tnf*, *Il6*, *Il12b*, *Il2*, *Ifng*, *Il1b*, and *Il18*, while decreasing the levels of anti‐inflammatory cytokine genes, including *Il4*, *Il5* and *Tgfb1*, indicative of a shift from Th2 toward Th1 immune response (Figure 4f).

Several investigations have demonstrated that RIG‐I agonists greatly induce synergistic anti‐tumour effects when combined with immune checkpoint inhibitors, such as anti‐CTLA‐4 and anti‐PD‐1 antibodies (Heidegger et al., 2019; Jiang et al., 2019; Ruzicka et al., 2020). Given its success in melanoma and non‐small cell lung cancer models, we sought to examine whether anti‐PD‐L1 antibody in combination with immRNA might enhance the anti‐tumour efficacy of single treatment in mouse breast cancer model. To this end, we treated 4T1 tumours with 2.5 mg/kg immRNA‐loaded RBCEVs (i.t) and 2 mg/kg anti‐PD‐L1 monoclonal antibody (i.p.) one day apart. Monotherapy of immRNA‐loaded RBCEVs or anti‐PD‐L1 antibody was used as a control. The dose and frequency of anti‐PD‐L1 antibody treatment was selected based on a previous study (Hong et al., 2019). 2 mg/kg of anti‐PD‐L1 is 34 times lower than the clinical‐equivalent dose (Herbst et al., 2014). Surprisingly, we observed that two out of four mice treated with anti‐PD‐L1 alone, and two out of four mice treated with immRNA‐RBCEVs combined with anti‐PD‐L1 were dead after three or five doses (Figure S4B). In contrast, all of the mice survived after five doses of immRNA‐RBCEVs monotherapy treatment (Figure S4B). The serum IFNβ level of the survived mice at the endpoint suggested that anti‐PD‐L1 antibody alone triggered cytokine release syndrome, which seemed to be alleviated when anti‐PD‐L1 was combined with immRNA‐RBCEVs even though the endpoint‐related mortality was the same as that caused by anti‐PD‐L1 monotherapy (Figure S4D). The combined treatment and anti‐PD‐L1 treatment alone did not have an improved effect on tumour suppression as compared to immRNA‐RBCEVs monotherapy (Figure S4C). Therefore, these data suggest that immRNA‐loaded RBCEVs are safe and sufficiently effective as a single‐agent immunotherapy for breast cancer treatment.

As a model of human breast cancer, we implanted CA1a cells in the fourth MFP of immunocompromised NSG‐SGM3 mice (Figure 4i). Similarly, we treated the tumours with immRNA‐loaded RBCEVs intratumorally every three days and measured the tumour size (Figure 4i). The treatment with immRNA‐loaded RBCEVs inhibited the growth of CA1a tumours significantly (Figure 4j). qPCR results showed that the RIG‐I cascade including *DDX58*, *MDA5*, *MAVS*, *IRF3*, *IRF7*, *IFNB*, *RSAD2*, and *ISG56* was up‐regulated in the tumours upon immRNA‐RBCEVs treatment (Figure 4k). Additionally, the treatment with immRNA‐RBCEVs was associated with enhanced infiltration of neutrophils, macrophages and dendritic cells in the tumours (Figures 4l and S4E). The same set of experiments was carried out in the MDA‐MB‐468 tumour model in nude mice, with four mice per group. immRNA‐loaded RBCEVs treatment led to rapid and extensive shrinkage of MDA‐MB‐468 tumours (Figure S4F), one of which was eliminated. We were only able to address RIG‐I activation with the limited number of tumour cells by qPCR. The results revealed that immRNA‐loaded RBCEVs treatment activated the RIG‐I pathway by up‐regulating *DDX58*, *MDA5*, *RSAD2*, *MAVS*, *IRF3*, *IRF7*, *IFNB*, and *ISG56* in the tumour cells (Figure S4G). Taken together, these data suggest that intratumoral administration of immRNA‐RBCEVs triggers anti‐tumour responses by activating the RIG‐I pathway and recruiting immune cells for orthotopic and xenograft breast cancer suppression.

### 2.5 Intratumoral delivery of bi‐functional ASO suppresses breast cancer growth by triggering apoptosis and RIG‐I mediated immune responses

**FIGURE 5:**
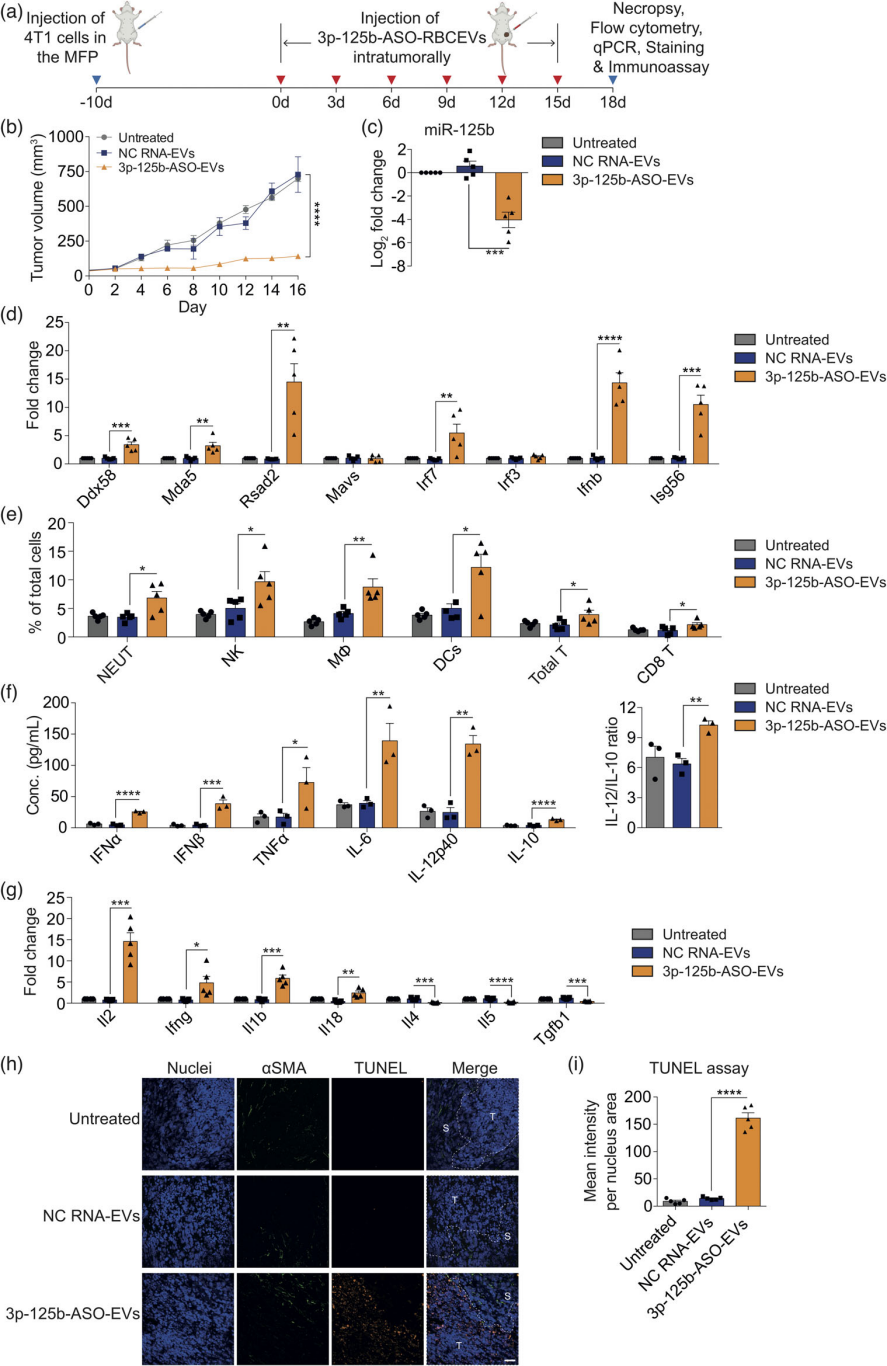
Intratumoral delivery of bi‐functional ASO suppresses breast cancer growth by triggering apoptosis and RIG‐I mediated immune responses. (a) Schematic treatment of mouse 4T1 mammary tumours in BALB/c mice with intratumoral delivery of 3p‐125b‐ASO in RBCEVs. (b) Volume of 4T1 tumours injected intratumorally with 5 mg/kg RBCEVs containing 3p‐125b‐ASO or NC RNA every three days (*n* = 5 mice). (c) qPCR analysis of miR‐125b relative to *sno234* in untreated and treated 4T1 tumours (*n* = 5 mice). (d) qPCR analysis of RIG‐I pathway gene expression relative to *Gapdh* in untreated and treated 4T1 tumours (*n* = 5 mice). (e) Flow cytometry analysis of immune cells in untreated and treated 4T1 tumours (*n* = 5 mice). NEUT, neutrophils; NK, natural killer cells; MΦ, macrophages; DCs, dendritic cells. (f) Multiplex immunoassay analysis of cytokine concentration (conc.) in the tumour lysates of mice (*n* = 3 mice). (g) qPCR analysis of cytokine gene expression relative to *Gapdh* in untreated and treated 4T1 tumours (*n* = 5 mice). (h) Representative images of TUNEL staining (orange fluorescence) of untreated and treated 4T1 tumour sections. Cancer‐associated fibroblasts (green) were stained with anti‐αSMA antibody. Nuclei were stained with Hoechst (blue). Scale bar, 50 μm. T, Tumour; S, Stroma. (i) Average mean intensity per nucleus area of TUNEL staining signals (*n* = 4 mice). All bar graphs represent mean ± SEM. *p < 0.05, **p < 0.01, ***p < 0.001, and ****p < 0.0001 determined by Student's two‐tailed *t*‐test

Next, we confirmed the anti‐tumour efficacy of 3p‐125b‐ASO in 4T1 cancer model in vivo. 4T1 cells were implanted in the fourth MFP of BALB/c mice (Figure 5a). 4T1 tumours were treated with 5 mg/kg 3p‐125b‐ASO‐loaded RBCEVs every three days and monitored every two days (Figure 5a). The mice were sacrificed and tumours were collected when the untreated tumours reached ∼15 mm in diameter. Similar to the immRNA treatment, we observed that 3p‐125b‐ASO delivered using RBCEVs suppressed 4T1 tumour growth significantly (Figure 5b). qPCR analysis of the dissociated tumour cells clearly showed decreased level of miR‐125b and increased levels of *Ddx58, Mda5, Irf7*, *Ifnb*, *Rsad2*, and *Isg56* in the tumours treated with 3p‐125b‐ASO‐loaded RBCEVs as compared to NC‐RNA‐loaded RBCEVs (Figure 5c‐d). Furthermore, flow cytometry analysis revealed increased numbers of tumour‐infiltrating neutrophils, NK cells, macrophages, dendritic cells, total T cells, and CD8^+^ T cells in the tumours treated with 3p‐125b‐ASO‐loaded RBCEVs as compared with NC‐RNA‐loaded RBCEVs (Figures 5e and S5). As only a low systemic IFNβ level was detected by ELISA in the tumour‐bearing mice receiving intratumoral administration of immRNA‐RBCEVs, we performed cytokine immunoassay in the tumour lysates to improve the sensitivity in detecting the lowest concentration of cytokines. The data revealed that elevated levels of IFNα (∼25.62 pg/ml), IFNβ (∼38.71 pg/ml), TNFα (∼72.64 pg/ml), IL‐6 (∼139.26 pg/ml), IL‐12p40 (∼100.78 pg/ml), and IL‐10 (∼23.06 pg/ml) were detected in the tumours treated with 3p‐125b‐ASO‐loaded RBCEVs as compared to the controls (Figure 5f). The IL‐12/IL‐10 ratio significantly increased upon 3p‐125b‐ASO‐loaded RBCEV treatment (Figure 5f), suggesting a pro‐inflammatory tumour microenvironment. An additional qPCR analysis of the tumour cells showed significant increases in *Il2*, *Ifng*, *Il1b*, and *Il18*, and decreases in *Il4*, *Il5*, and *Tgfb1* (Figure 5g), indicative of Th1‐dominant immune response. Indeed, 3p‐125b‐ASO‐loaded RBCEVs substantially induced apoptosis with TUNEL positive cells concentrated in the tumour bulk but not in the stroma as determined by TUNEL staining of the tumour sections, suggesting that miR‐125b knockdown and RIG‐I activation contribute to the tumoricidal activity mediated by 3p‐125b‐ASO (Figure 5h‐i). Together, these data provide evidence that both miR‐125b inhibition and RIG‐I‐mediated activation enhanced the anti‐tumour efficacy of 3p‐125b‐ASO in an orthotopic breast cancer model.

### 2.6 Conjugation of RBCEVs with EGFR‐binding nanobody promotes specific delivery of immRNA to metastatic EGFR‐positive breast cancer cells

**FIGURE 6:**
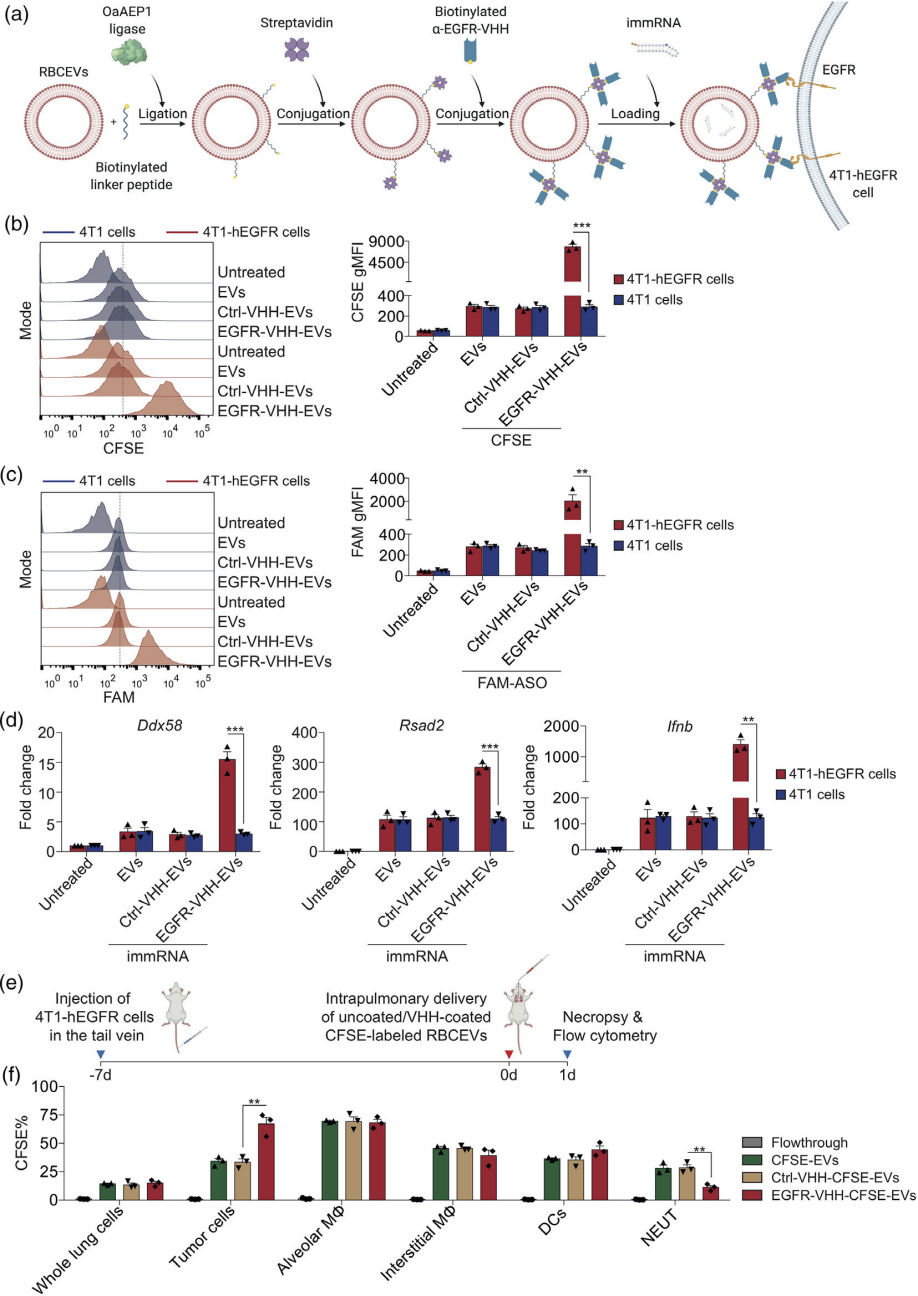
Conjugation of RBCEVs with EGFR‐binding nanobody promotes specific delivery of immRNA to metastatic breast cancer cells. (a) Schematic RBCEV modification: RBCEVs were conjugated with an anti‐EGFR nanobody and loaded with immRNA. (b) Flow cytometry analysis of 4T1‐hEGFR cells and parental 4T1 cells treated with 0.02 μg/μl uncoated, control (Ctrl)‐VHH‐coated and EGFR‐VHH‐coated CFSE‐labelled RBCEVs (*n* = 3). (c) Flow cytometry analysis of 4T1‐hEGFR cells and parental 4T1 cells treated with 0.02 μg/μl uncoated, Ctrl‐VHH‐coated and EGFR‐VHH‐coated FAM‐NC‐ASO‐loaded RBCEVs (*n* = 3). (d) qPCR analysis of the RIG‐I pathway gene expression relative to *Gapdh* in 4T1‐hEGFR cells and parental 4T1 cells treated with 0.1 μg/μl uncoated, Ctrl‐VHH‐coated and EGFR‐VHH‐coated RBCEVs containing immRNA (*n* = 3). (e) Schematic intrapulmonary delivery of EGFR‐targeted CFSE‐RBCEVs in mice bearing metastatic 4T1‐hEGFR tumours. (f) Flow cytometry analysis of CFSE signals in tumour cells and immune cells in the lungs of mice bearing 4T1‐hEGFR tumours treated with 25 mg/kg uncoated, Ctrl‐VHH‐coated and EGFR‐VHH‐coated CFSE‐RBCEVs (*n* = 3 mice). NEUT, neutrophils; MΦ, macrophages; DCs, dendritic cells. All bar graphs represent mean ± SEM. **p < 0.01 and ***p < 0.001 determined by Student's two‐tailed *t*‐test

Targeting cancer cells specifically is a vital characteristic of EV‐based drug delivery, as it enhances the therapeutic efficacy while protecting normal cells from toxicity. To this end, we sought to deliver immRNA‐loaded RBCEVs specifically to the cancer cells in lung metastatic breast cancer models. Epidermal growth factor receptor (EGFR), a proto‐oncogene in many types of cancers, is considered a common cancer biomarker (Normanno et al., 2006). We transduced mouse breast cancer 4T1 cells with lentiviruses carrying the human EGFR expression vectors. We had previously developed an RBCEV surface functionalization method using OaAEP1 ligase to conjugate peptides with ligase‐binding motif (NGL) at the C‐terminus onto the RBCEV surface (Pham et al., 2021). In the present study, we combined the enzymatic ligation method with a streptavidin‐biotin conjugation method to conjugate RBCEVs with nanobodies via a linker peptide (Figure 6a). Specifically, RBCEVs were enzymatically ligated with a biotinylated linker peptide (biotin‐TRNGL), which was sequentially conjugated with tetrameric streptavidin and biotinylated anti‐EGFR nanobody (α‐EGFR‐VHH) (Figure 6a). Flow cytometry analysis showed the specific binding of α‐EGFR‐VHH to human EGFR‐expressing cells (CA1a, H358, and 4T1‐hEGFR cells) rather than mouse EGFR‐expressing 4T1 cells (Figure S1C). To assess the specificity of EGFR‐targeting RBCEVs towards EGFR‐expressing cancer cells, EGFR‐VHH‐coated RBCEVs were labelled with CFSE and incubated with 4T1 cells expressing human EGFR (4T1‐hEGFR) and parental 4T1 cells at a suboptimal dose for 2 h. An anti‐mCherry nanobody was used as a negative control (Ctrl‐VHH). The CFSE fluorescence intensity in 4T1‐hEGFR cells treated with EGFR‐targeting CFSE‐labelled RBCEVs was ∼28.6‐fold higher than that in 4T1‐hEGFR cells treated with non‐targeted CFSE‐labelled RBCEVs (Figure 6b). The non‐targeted CFSE‐labelled RBCEVs were taken up by 4T1‐hEGFR cells at similar levels as non‐targeted and EGFR‐targeted CFSE‐labelled RBCEVs by 4T1 cells (Figure 6b). We further assessed the specific uptake of EGFR‐targeting RBCEVs carrying fluorescent RNAs in 4T1‐hEGFR cells. Consistently, the FAM fluorescence intensity was ∼7.5‐fold higher in hEGFR‐positive 4T1 cells treated with EGFR‐VHH‐coated FAM‐ASO‐loaded RBCEVs compared to parental 4T1 cells treated with EGFR‐VHH‐coated FAM‐ASO‐loaded RBCEVs, and 4T1‐hEGFR cells treated with non‐targeted FAM‐ASO‐loaded RBCEVs (Figure 6c). To verify if the EGFR‐targeting RBCEVs specifically deliver functional RNAs to 4T1‐hEGFR cells, we loaded EGFR‐VHH‐coated RBCEVs with immRNA. As expected, EGFR‐VHH‐coated immRNA‐loaded RBCEVs exhibited a higher functional uptake by 4T1‐hEGFR cells compared to 4T1 cells as evidenced by the substantial increases in cellular *Ddx58*, *Rsad2*, *Ifnb, Mda5, Mavs, Irf3, Irf7*, and *Isg56* expression (Figures 6d and S6A).

To generate a lung metastatic breast cancer model, we injected 4T1‐hEGFR cells intravenously in the tail vein of BALB/c mice (Figure 6e). After seven days, we delivered non‐targeted and EGFR‐targeted CFSE‐labelled RBCEVs via intrapulmonary administration to the mice bearing metastatic breast cancer. CFSE‐labelled RBCEVs were washed extensively using size exclusion chromatography and centrifugation. The flowthrough of the last wash was used as a negative control. Twenty‐four hours after intrapulmonary delivery, the biodistribution of non‐targeted and EGFR‐targeted CFSE‐labelled RBCEVs in the lungs was analyzed using flow cytometry. The intrapulmonary delivery of EGFR‐targeted and non‐targeted RBCEVs was consistent as shown by a similar percentage (∼16%) of CFSE‐positive cells in the homogenized whole lung cells (Figures 6f and S6B). Importantly, the conjugation of RBCEVs with EGFR nanobody increased the percentage of CFSE‐positive tumour cells to ∼68% (Figures 6f and S6B). No significant change was observed in alveolar macrophages, which are located in the airway lumen and always take up EVs at a high rate (Figures 6f and S6B). Besides, we also observed a significant decrease in the percentage of CFSE‐positive neutrophils and a slight decrease in the percentage of CFSE‐positive interstitial macrophages in the lungs of mice treated with EGFR‐targeting RBCEVs (Figures 6f and S6B). This can be attributed to the hEGFR‐specific RBCEVs being preferentially taken up by hEGFR‐positive tumour cells over the hEGFR‐negative cells in the lung parenchyma. These data suggest that EGFR nanobody conjugation can drive RBCEVs specifically towards EGFR‐positive metastatic breast cancer cells in vivo.

### 2.7 Intrapulmonary delivery of immRNA using EGFR‐targeted RBCEVs suppresses breast cancer metastasis in the lung

**FIGURE 7:**
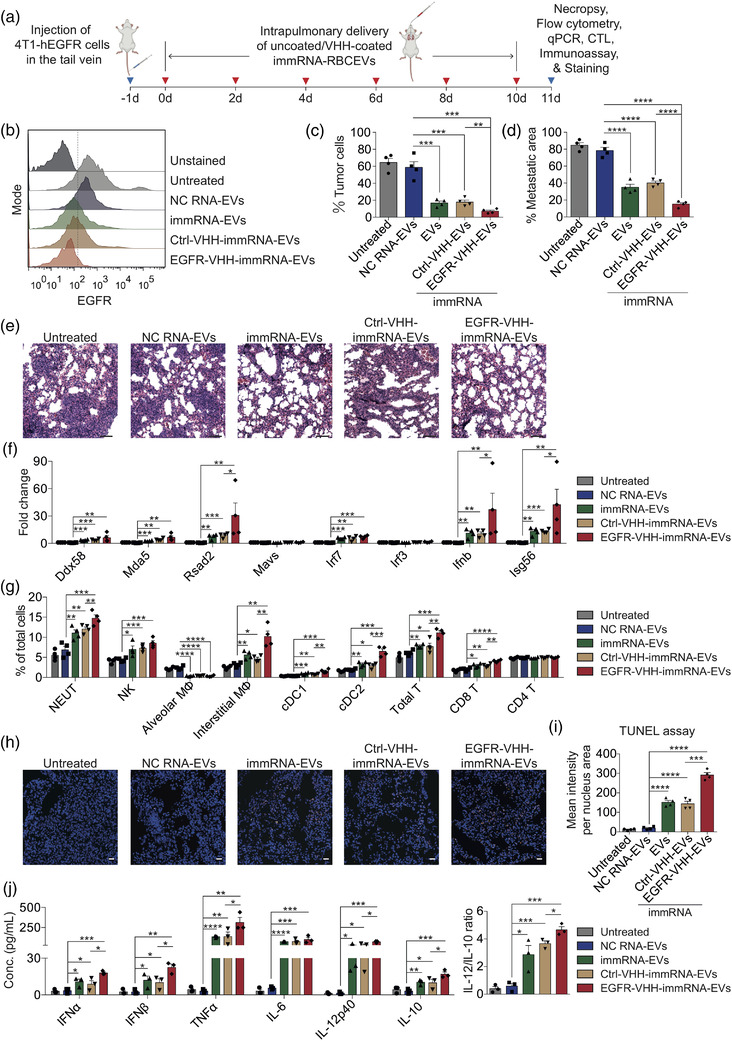
Intrapulmonary delivery of immRNA using EGFR‐targeted RBCEVs suppresses breast cancer metastasis in the lung. (a) Schematic intrapulmonary treatments for mice bearing breast cancer metastasis. (b) Flow cytometry analysis of tumour burden in the lungs using anti‐human‐EGFR antibody after five treatments. (c) Average percentage of hEGFR‐positive tumour cells in the lungs as shown in (b) (*n* = 4 mice). (d) Average percentage of metastatic area in the lungs treated with 25 mg/kg uncoated, Ctrl‐VHH‐coated and EGFR‐VHH‐coated RBCEVs containing immRNA after five treatments (*n* = 4 mice). (e) Representative H & E stained lung sections from mice as in (d). Scale bar, 50 μm. (f) qPCR analysis of the RIG‐I pathway gene expression relative to *Gapdh* in the lungs of mice as in (a) (*n* = 4 mice). (g) Flow cytometry analysis of immune cells in the lungs of mice as in (a) (*n* = 4 mice). (h) Representative images of TUNEL staining (orange fluorescence) of lung sections from mice as in (a). Nuclei were stained with Hoechst (blue). Scale bar, 20 μm. (i) Average mean intensity per nucleus area of TUNEL staining signals (*n* = 4 mice). (j) Multiplex immunoassay of cytokines in the lung homogenates of mice with 4T1‐hEGFR metastatic tumours (*n* = 3 mice). All bar graphs represent mean ± SEM. *p < 0.05, **p < 0.01, ***p < 0.001, and ****p < 0.0001 determined by Student's two‐tailed *t*‐test

In order to treat metastatic breast cancer in the lung, we delivered multiple doses of EGFR‐targeting immRNA‐loaded RBCEVs via intrapulmonary administration every other day (Figure 7a). After five treatments, the mice were sacrificed and the lungs were excised for subsequent analysis. As expected, non‐targeted and EGFR‐targeted immRNA‐loaded RBCEVs suppressed the engraftment of metastatic tumour cells as shown by the decreased percentage of hEGFR‐positive cells in the lungs (Figures 7b‐c and S7A). In particular, EGFR‐targeted immRNA‐loaded RBCEVs showed greater tumour suppression in the lungs, an effect attributed to the increased immRNA delivery to tumour cells in this treatment. H & E staining of the lung sections showed that the metastatic areas of lungs were significantly lower in the mice treated with EGFR‐targeted immRNA‐loaded RBCEVs (Figure 7d‐e). qPCR analysis of the whole lung lysate revealed that immRNA‐loaded RBCEVs triggered RIG‐I cascade activation by up‐regulating *Ddx58*, *Mda5*, *Rsad2*, *Irf7*, *Ifnb*, and *Isg56*. Although there was no substantial increase in RLR genes, *Ifnb* and IFN‐stimulated genes *Isg56* and *Rsad2* increased significantly upon EGFR‐targeted RBCEVs treatment compared to non‐targeted RBCEVs treatment (Figure 7f). According to flow cytometry analysis, increased infiltrations of neutrophils, NK cells, interstitial macrophages, cDCs, T cells including CD8^+^ T cells but not CD4^+^ T cells, were observed in the lungs of mice treated with immRNA‐loaded RBCEVs (Figures 7g and S7A). EGFR‐targeted RBCEVs further increased the number of infiltrating neutrophils, interstitial macrophages, cDCs, T cells and CD8^+^ T cells (Figures 7g and S7A). Alveolar macrophages were mostly eliminated in the airways of mice treated with the control and EGFR‐targeted RBCEVs containing immRNA (Figures 7g and S7A). TUNEL staining of the lung sections further confirmed that the treatment with EGFR‐targeted RBCEVs containing immRNA increased apoptosis in the lungs compared to the treatment with non‐targeted immRNA‐loaded RBCEVs (Figure 7h‐i). To investigate whether the respiratory inflammatory state in mice affects metastasis, we detected the concentrations of cytokines in the lung homogenates. All tested cytokines were significantly elevated in the lungs of mice treated with EGFR‐targeted and non‐targeted immRNA‐loaded RBCEVs versus the control mice (Figure 7j). The mice receiving EGFR‐targeted immRNA‐loaded RBCEVs exhibited even higher IFNα (∼17.97 pg/ml), IFNβ (∼22.47 pg/ml), TNFα (∼312.86 pg/ml), IL‐12p40 (∼72.73 pg/ml), IL‐10 (∼16.91 pg/ml), and IL‐12/IL‐10 ratio, as compared with those receiving non‐targeted immRNA‐loaded RBCEVs, whereas there was no significant difference in the levels of IL‐6 (Figure 7j). qPCR analysis of the lung lysate revealed that immRNA‐loaded RBCEVs increased the mRNA levels of *Il2*, *Ifng*, *Il1b*, and *Il18*, while decreasing the levels of *Il4*, *Il5*, and *Tgfb1* (Figure S7B), indicating Th1‐dominant immunity in the lung. EGFR‐targeted RBCEVs further enhanced the expression of *Il2* and *Ifng*, and reduced the expression of *Tgfb1*, as compared to non‐targeted RBCEVs (Figure S7B). Taken together, these data suggest that EGFR‐targeted RBCEVs enhance the specificity of delivery to EGFR‐positive cancer cells leading to improved therapeutic efficacy of immRNA in vivo.

### 2.8 EGFR‐targeted immRNA‐loaded RBCEVs induce DC activation, promote M1 macrophage polarization and potentiate tumour‐specific CD8^+^ T cell activity

**FIGURE 8:**
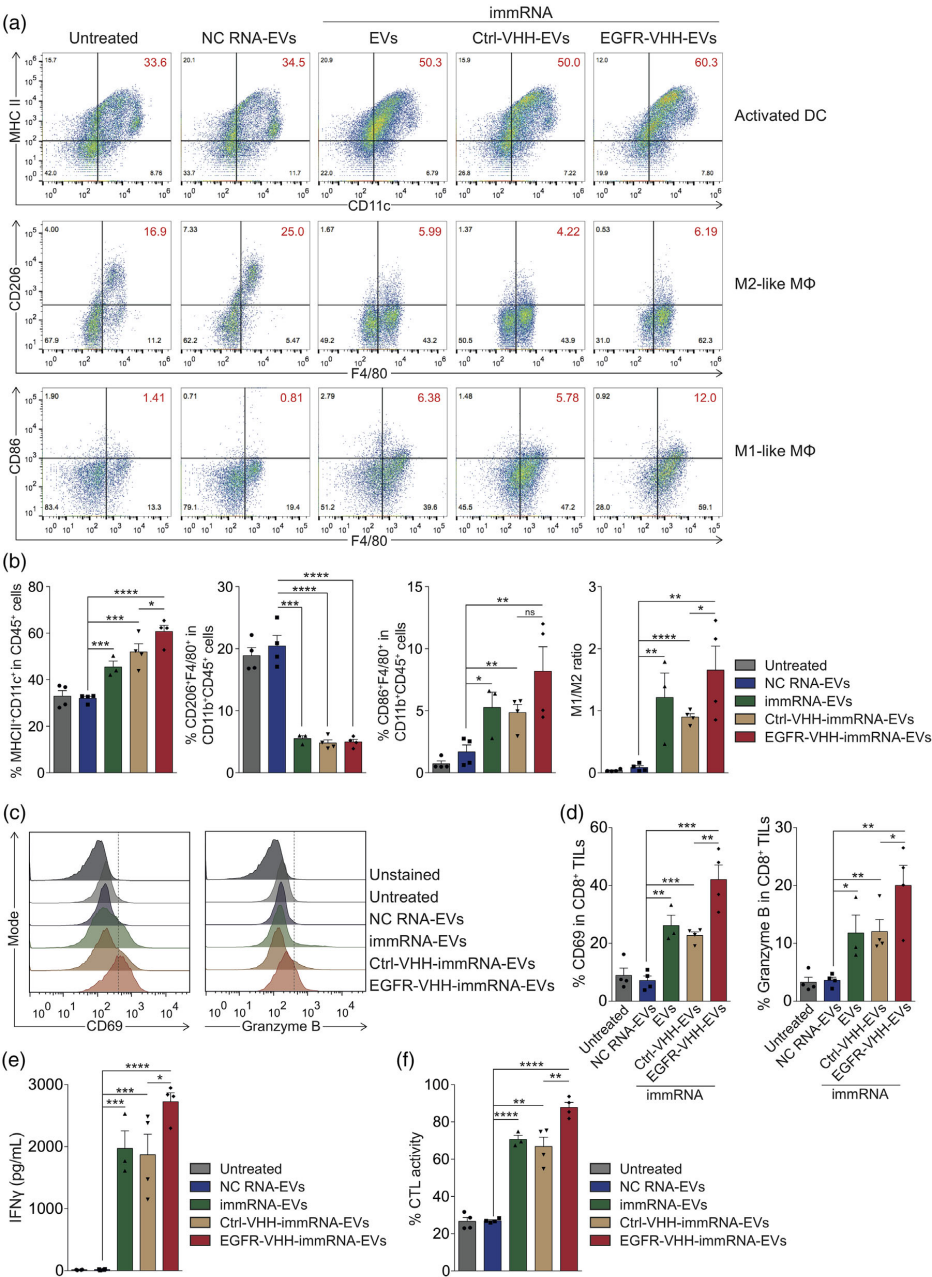
EGFR‐targeted immRNA‐loaded RBCEVs induce DC activation, promote M1 macrophage polarization and potentiate tumour‐specific CD8^+^ T cell activity. (a) Flow cytometry analysis of activation and polarization of DCs and macrophages in the lungs of mice as in Figure 7a (*n* = 3–4 mice). MΦ, macrophages. (b) Average percentage of cells as shown in (a) (*n* = 3–4 mice). (c) Flow cytometry analysis of CD69 and granzyme B in CD8^+^ tumour‐infiltrating lymphocytes (TILs) from the lungs of mice as in Figure 7a (*n* = 3–4 mice). (d) Average percentage of CD69 and granzyme B in CD8^+^ TILs as shown in (c). (e) ELISA quantification of IFNγ from tumour‐specific CD8^+^ TILs in the culture supernatants at 48 h (*n* = 3–4 mice). (f) Tumour‐specific cytotoxic T lymphocyte (CTL) activity of CD8^+^ TILs (*n* = 3–4 mice). All bar graphs represent mean ± SEM. ns, not significant; *p < 0.05, **p < 0.01, ***p < 0.001, and ****p < 0.0001 determined by Student's two‐tailed *t*‐test

To assess the ability of EGFR‐targeted immRNA‐loaded RBCEVs to induce DC activation and regulate macrophage polarization in the lung, the expression of DC activation marker and macrophage M1/M2 markers were determined using flow cytometry. The data revealed that non‐targeted and EGFR‐targeted immRNA‐loaded RBCEVs treatments elevated the percentage of MHCII^+^CD11c^+^ DCs in CD45^+^ cells (Figure 8a‐b). EGFR‐targeted immRNA‐loaded RBCEVs treatment further increased the percentage of MHCII^+^CD11c^+^ DCs as compared to non‐targeted immRNA‐loaded RBCEVs treatment (Figure 8a‐b). In addition, immRNA‐loaded RBCEVs treatment led to lower percentage of CD206^+^F4/80^+^ M2‐like macrophages and higher percentage of CD86^+^F4/80^+^ M1‐like macrophages in CD11b^+^CD45^+^ cells (Figure 8a‐b). The M1/M2 ratio significantly increased upon EGFR‐targeted immRNA‐loaded RBCEVs treatment as compared to non‐targeted immRNA‐loaded RBCEVs treatment (Figure 8b).

To quantify the activity of CD8^+^ tumour‐infiltrating lymphocytes (TILs) against tumour‐associated antigens, we isolated CD8^+^ TILs from the lungs for cytotoxicity analysis. As a result, CD69 and granzyme B levels significantly increased in CD8^+^ TILs upon immRNA‐loaded RBCEV treatments (Figure 8c‐d). Given that the tumour‐specific immune response is a key factor in cancer immunotherapy, we next evaluated the tumour‐specific T cell response. We incubated CD8^+^ TILs with 4T1‐hEGFR‐luciferase cells at a ratio of 50:1 for 48 h. Re‐stimulation of isolated CD8^+^ TILs from immRNA‐loaded RBCEVs treated mice with tumour antigens markedly augmented the production of IFNγ, which is associated with a significant increase in cytotoxic T lymphocytes (CTL) activity (Figure 8e‐f). EGFR‐targeted immRNA‐loaded RBCEVs further potentiated the cytotoxicity of CD8^+^ TILs as demonstrated by the increased cellular expression of CD69 and granzyme B, secretion of IFNγ and CTL activity (Figure 8c‐f). Collectively, these data correlated with the elevated expression of Th1 cytokines and reduced expression of Th2 cytokines in the lungs of EGFR‐targeted immRNA‐RBCEVs treated mice (Figures 7j and S7B), indicating Th1‐associated and CTL‐mediated anti‐tumour immune responses.

## 3 DISCUSSION

**FIGURE 9:**
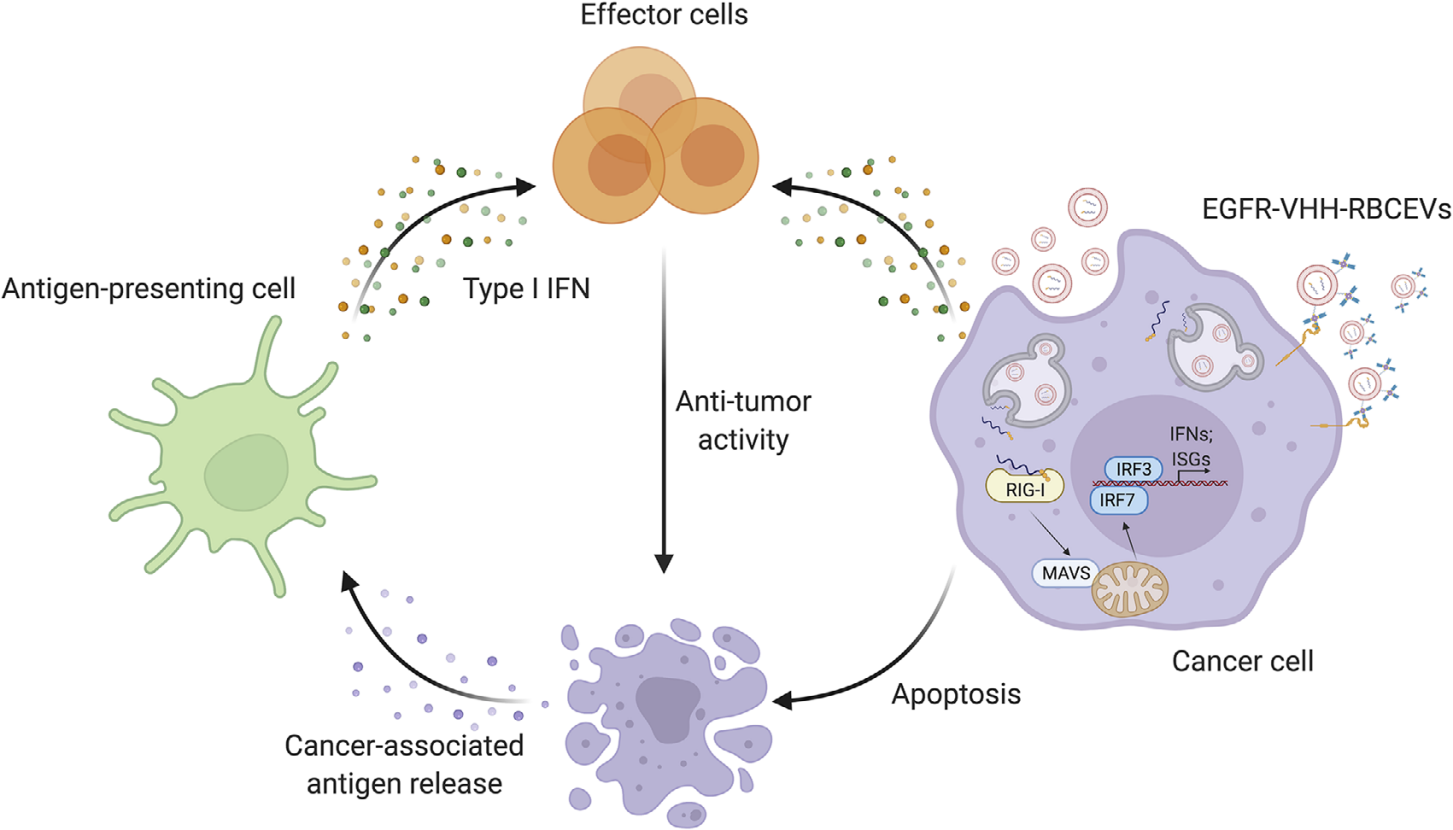
Schematic delivery of RIG‐I agonists using RBCEVs for anti‐cancer immunotherapy

In summary, we have demonstrated here a robust platform for local and targeted delivery of immunomodulatory RIG‐I agonists using RBCEVs. The treatments of orthotopic and metastatic breast cancers with both immRNA‐ and 3p‐125b‐ASO‐loaded RBCEVs allowed activation of RIG‐I signalling, which results in a three‐pronged attack: (i) strong tumour growth inhibition; (ii) augmented levels of type I IFN and pro‐inflammatory cytokines; and (iii) IFN‐mediated recruitment of innate immune cells (macrophages, neutrophils, NK cells), activation of dendritic cells and M1 macrophage polarization, and cross‐priming of adaptive immune effectors (CD8^+^ T cells) through antigen‐presenting cells (dendritic cells and macrophages) (Figure 9), suggesting a reversal of the immunosuppressive tumour microenvironment. Polarization of Th1/Th2 immune factors promotes anti‐tumour immunocompetence. Th1 cytokines, such as IL‐1β, IL‐2, IL‐12, IL‐18, TNFα, and IFNγ, are associated with proinflammation, while Th2 cytokines, such as IL‐4, IL‐5, and TGFβ, play a suppressive role in the tumour immune microenvironment (Grivennikov et al., 2010; Zhao et al., 2021). Our data demonstrate that immRNA‐ and 3p‐125b‐ASO‐loaded RBCEVs altered the Th1/Th2 balance toward Th1 dominance in the tumour microenvironment contributing to tumour suppression. These findings are consistent with prior studies where shifting the tumour microenvironment to Th1 dominance has beneficial effects on anti‐tumour immunity (Hayata et al., 2013; Li et al., 2017). Effective cancer immunotherapy requires T cell priming, activation and tumour infiltration. Our data also demonstrate that immRNA‐loaded RBCEVs promoted CD8^+^ T cell responses to tumour antigens accompanied by Th1 polarization and tumour‐specific CTL activity in the metastatic tumour model. In addition, non‐malignant mammary gland epithelial cells and lung epithelial cells are likely protected by some mechanisms from apoptosis after the treatment with immRNA‐ and 3p‐125b‐ASO‐loaded RBCEVs (Besch et al., 2009). Hence, intratumoral delivery of immRNA‐ and 3p‐125b‐ASO‐loaded RBCEVs induced massive cell apoptosis in the tumour bulk, whereas tumour stroma showed no signs of apoptosis. These findings are supported by a previous report, which demonstrated that tumour cells are highly susceptible to RIG‐I‐mediated apoptosis (Besch et al., 2009).

RIG‐I agonists in combination with immune checkpoint inhibitors anti‐PD‐1 and anti‐CTLA‐4 have proven to be effective as a combined therapy for melanoma and AML (Heidegger et al., 2019; Jiang et al., 2019; Ruzicka et al., 2020). In this study, we sought to combine immRNA‐loaded RBCEVs and anti‐PD‐L1 to achieve enhanced anti‐tumour activity. However, we observed ∼50% mortality in tumour‐bearing mice receiving repeated doses of systemic anti‐PD‐L1 administration. The fatality of mice might be attributed to the hypersensitivity reactions caused by anti‐PD‐L1 (Mall et al., 2016). These findings highlight the previously uncharacterized adverse toxicity of anti‐PD‐L1 not reported in previous studies of RIG‐I‐agonist and anti‐PD‐L1 combination. Indeed, various clinical studies have demonstrated that anti‐PD‐L1 as a single treatment greatly induced systemic IFNβ release and cytokine storm in agreement with our observation (Delanoy et al., 2019; Michot et al., 2018). Systemic administration of RIG‐I agonists is likely to trigger cytokine release syndrome as well but local administration of immRNA is safe and sufficiently effective according to our data.

In this study, we have also demonstrated a combinatorial approach to simultaneously inhibit oncogenic miR‐125b and activate the RIG‐I pathway. Due to their genetic and epigenetic plasticity, tumour cells tend to evade single‐targeted therapies such as specific kinase/oncogene inhibitors or immunotherapies (Rubin et al., 2007). Therefore, multi‐targeted therapies are needed. 3p‐125b‐ASO offers advantages over combinations of multiple single‐targeted therapies. It is small, easy to synthesize and comprises two distinct and independent properties as a RIG‐I activator and an oncogene suppressor. Moreover, RIG‐I‐induced apoptosis of cancer cells is synergized with apoptosis induced by ASO‐mediated inhibition of miR‐125b. Such bi‐functional ASOs can be adapted to different tumour entities by targeting key oncogenes that drive tumorigenicity.

Collectively, these data suggest the potential of immRNA and 3p‐125b‐ASO for clinical application. Indeed, several clinical trials based on RIG‐I activation have reached the attention of pharmaceutical companies. A clinical study (NCT03065023) employing a RIG‐I agonist, termed MK‐4621 (RGT100), evaluated its safety, tolerability and anti‐tumour activity in patients with advanced or recurrent solid tumours. Intratumoral/intralesional administration of the synthetic RNA oligonucleotide via in vivo‐jetPEI delivery system resulted in no dose‐limiting toxicity and increased circulating chemokine levels in patients (Middleton et al., 2018). However, this trial has been withdrawn for unknown reasons. Another phase I dose‐escalation clinical trial (NCT02829723) is currently underway using RIG‐I agonist BLZ945 as monotherapy or in combination with PDR001, an anti‐PD‐1, in patients with advanced solid tumours. This trial, estimated to be completed upon March 2022, is designed to investigate the safety, tolerability, pharmacokinetics, pharmacodynamics and anti‐tumour activity of BLZ945, administered orally, as a single agent or in combination with PDR001, administered intravenously. Preclinical study of BLZ945 is not yet published hence it is unclear how BLZ945 is delivered orally and if this delivery method is effective.

Biosafety and reproducibility of drug delivery systems are critical for their clinical translation. In vivo‐jetPEI is a hitherto widely used delivery system for RIG‐I agonists and other kinds of RNA therapeutics in preclinical and clinical studies. However, it has been reported to be associated with fatal hepatotoxicity in mice (Akita et al., 2011; Hayashi et al., 2012). A research group utilized lipid‐based nanoparticles to deliver bi‐functional siRNA for cancer treatment (Das et al., 2019). Although the toxicity of nanoparticles was barely noticeable, off‐target effects were observed with higher accumulation in fibroblasts over cancer cells in the tumours (Das et al., 2019). Owing to their biocompatibility, stability and limited immunogenicity, EVs provide multiple advantages as a delivery system over traditional synthetic delivery vehicles. RBCEVs with high availability, scalability and without risk of horizontal gene transfer have been illustrated to be safe, efficient and amenable for therapeutic delivery in cancer treatment (Pham et al., 2021; Usman et al., 2018). In the present study, we achieved effective immunomodulation of the tumour microenvironment and potent anti‐tumour activity using RBCEVs as delivery vehicles for RIG‐I agonists without any observable adverse effects, suggesting the advantages of RBCEVs for clinical application.

We further described an RBCEV surface functionalization method with EGFR‐targeted nanobodies, which can enhance the delivery of RIG‐I agonists toward EGFR‐positive cancer cells, thereby improving therapeutic efficacy while reducing side effects. In our experiments, a depletion of alveolar macrophages in the respiratory tract was observed upon intrapulmonary delivery of non‐targeted and EGFR‐targeted RBCEVs containing immRNA. Another research group has previously reported the same phenomenon when delivering drug‐loaded liposomes to the lung (Thepen et al., 1989; Worgall et al., 1997). From our data on biodistribution of intrapulmonary delivery of RBCEVs, alveolar macrophages were the primary recipient of both non‐targeted and EGFR‐targeted RBCEVs. It is evident that RBCEVs themselves have no influence on alveolar macrophages because CFSE‐labelled or NC‐RNA‐loaded RBCEVs did not cause rapid death of these cells. The alveolar macrophages are assigned an important role in removing antigens in the lungs by nonspecific phagocytosis. The depletion of alveolar macrophages might be an effect attributed to RIG‐I‐mediated immune responses from phagocytized RBCEVs containing immRNA. However, the mechanism is still obscure. The depletion of alveolar macrophages may indeed increase the availability of RBCEVs, thus improving the efficiency of EGFR‐VHH‐coated immRNA‐loaded RBCEVs to target EGFR‐positive cancer cells in the lung parenchyma. To our knowledge, this is the first study demonstrating the EV‐mediated delivery of therapeutic RNA via intrapulmonary administration. Our data show that RNA‐loaded RBCEVs are stable and able to penetrate deeply into the lung parenchyma for the delivery of immRNA. This is a promising strategy for therapeutic RNA delivery that is applicable not only to cancer metastasis but also to other pulmonary diseases such as influenza and asthma.

## 4 MATERIALS AND METHODS

### 4.1 Cell culture

Mouse breast cancer 4T1 cell line and human breast cancer MCF10CA1a (CA1a) cell line were purchased from Karmanos Cancer Institute (Wayne State University, USA). Human breast cancer MDA‐MB‐468 and MDA‐MB‐231 cell lines, human untransformed mammary gland epithelial cell line MCF10A, and human lung cancer NCI‐H358 (H358) cell line were obtained from the American Type Culture Collection (ATCC, USA). Human lung epithelial carcinoma reporter cell lines A549‐Dual™ and A549‐Dual™ RIG‐I^−/−^ were purchased from InvivoGen, USA. All cells except MCF10A cells were cultured in Dulbecco's Modified Eagle Media—DMEM (Thermo Fisher Scientific, USA) supplemented with 10% fetal bovine serum—FBS (Biosera, France), 1 × penicillin‐streptomycin (Thermo Fisher Scientific), and 5 μg/ml Plasmocin™ prophylactic (InvivoGen, USA) in a humidified incubator at 37°C with 5% CO_2_. MCF10A cells were cultured in MEBM medium supplemented with the additives from the MEGM kit (Lonza, UK) and 100 ng/ml cholera toxin (Sigma, USA).

### 4.2 Mouse primary lung epithelial cell isolation

Mouse lung epithelial cells were isolated from female BALB/c mice as previously described (Kasinski & Slack, 2013). Briefly, 7‐week‐old mice were euthanized. Mouse lungs were excised and dissociated in RPMI media containing 10% FBS and 5 mg/ml collagenase IV (Thermo Fisher Scientific) using the GentleMACS dissociator (Miltenyi Biotec, Germany). Cells were filtered through a 70 μm strainer, washed and seeded in a 10‐cm plate. Fibroblasts are more sensitive to trypsin and can therefore be removed while the epithelial cells adhere for a longer time. Over the course of epithelial cell selection, removing the first cells that begin to slough off the plate upon trypsinization helps increase the purity of epithelial cells. EpCAM expression of epithelial cells was assessed using flow cytometry after three rounds of selection.

### 4.3 Purification of RBCEVs

Blood samples of healthy donors with informed consent were obtained from the Singapore Health Science Authority and Hong Kong Red Cross. RBCs were separated from whole blood and RBCEVs were purified from RBCs as described previously (Pham et al., 2021). Briefly, RBCs were separated from plasma using centrifugation at 1000*g* for 8 min at 4°C and washed three times with PBS (1000*g* for 8 min at 4°C). White blood cells were completely removed by leukodepletion filters (Nigale, China). Isolated RBCs were collected in Nigale buffer (0.2 g/L citric acid, 1.5 g/L sodium citrate, 7.93 g/L glucose, 0.94 g/L sodium dihydrogen phosphate, 0.14 g/L adenine, 4.97 g/L sodium chloride, 14.57 g/L mannitol), diluted in PBS containing 0.1 mg/ml calcium chloride, and incubated overnight with 10 μM calcium ionophore (Sigma, USA) at 37°C with 5% CO_2_. RBCs and cell debris were pelleted and the supernatant was collected by centrifugation at increasing speed (600*g* for 20 min, 1600*g* for 15 min, and 3260*g* for 15 min). The supernatant was filtered through a 0.45 μm membrane before ultracentrifugation at 50,000*g* for 70 min at 4°C using a SW32 rotor (Beckman Coulter, USA). The pellet was resuspended in 1 ml of PBS and subsequently loaded onto a 60% sucrose cushion and ultracentrifuged at 50,000*g* for 16 h at 4°C. RBCEVs were collected at the interface and washed with PBS at 50,000*g* for 70 min at 4°C. Purified RBCEVs were stored in PBS containing 4% trehalose at ‐80°C.

### 4.4 Characterization of RBCEVs

The size distribution and concentration of RBCEVs were quantified using a ZetaView^®^ nanoparticle tracking analyzer (Particle Metrix, Germany). Because hemoglobin is the major constituent of RBCEVs, RBCEV quantity is indicated by hemoglobin quantity throughout this study. The hemoglobin contents of RBCEVs were quantified using a NanoDrop™ 2000 spectrophotometer (Thermo Fisher Scientific).

### 4.5 Western blot analysis

RBCEVs were lysed with RIPA buffer (Thermo Fisher Scientific) supplemented with protease inhibitors (Biotool, USA) for 5 min on ice. Cells were lysed for 30 min on ice. Protein concentration was measured by a Pierce™ BCA assay (Life Technologies, USA) with BSA (New England Biolabs, UK) concentration as standards. A total of 50 μg protein lysate was loaded onto 10% polyacrylamide gels together with a Precision Plus Protein™ Kaleidoscope™ prestained protein standard (Bio‐Rad, USA). The proteins were transferred to Immobilon‐P polyvinylidene difluoride membranes (Merck Millipore, USA), which were blocked using 5% milk in Tris buffered saline containing 0.1% Tween‐20 (TBST) for 1 h followed by an incubation with primary antibody, anti‐ALIX antibody (Santa Cruz, USA, dilution 1:1,000), anti‐TSG101 antibody (Santa Cruz, dilution 1:1,000), anti‐human GAPDH antibody (Santa Cruz, dilution 1:1,000), anti‐human GPA antibody (Santa Cruz, dilution 1:500), anti‐human HBA antibody (Santa Cruz, dilution 1:1,000) or anti‐human β‐actin antibody (CST, USA, dilution 1:1,000) overnight at 4°C. The membranes were washed three times with TBST then incubated with HRP‐conjugated secondary antibody (Jackson ImmunoResearch, USA, dilution 1:5,000) for 1 h at room temperature. The blots were imaged using a ChemiDoc™ gel documentation system (Bio‐Rad).

### 4.6 Transmission electron microscopy

RBCEVs were fixed with 2% paraformaldehyde for 10 min and loaded on a glow‐discharged copper grid (200 mesh, coated with formvar carbon film). RBCEVs on the grid were incubated with 3% uranyl acetate for 5 min to perform negative staining of RBCEVs. This was followed by a quick wash with distilled water to remove excess stain. The grids were air dried for 10 min before being imaged using a Tecnai G2 transmission electron microscope (FEI) at 100 kV.

### 4.7 Single‐EV flow cytometry

Single‐EV flow cytometry was carried out using a CytoFLEX LX flow cytometer (Beckman Coulter, USA). RBCEVs were stained with anti‐human GPA‐FITC antibody (BioLegend, USA) for 3 h at 4°C, followed by two washing steps using sterile 0.2 μm‐filtered PBS to remove unbound antibody. We previously found that EV suspensions within the range of 3.9 × 10^3^ to 2.5 × 10^5^ EVs/μL allowed analysis of single EVs accurately (Pham et al., 2021). As such, the stained RBCEVs were diluted to a concentration of 3 × 10^4^ EVs/μl in 0.2 μm‐filtered PBS. RBCEVs were gated out from background noise using the violet side scatter (VSSC) channel acquired via the 405 nm laser (Figure S1B). Reference 100 nm beads were used to verify the gating of the EV population. Data was acquired using the following settings: FSC 138, SSC 180, FITC 3000, VSSC 800 with the threshold of the trigger signal (VSSC) set manually to 4000. The flow rate was maintained at 30 μl/min (achieving an event rate of ∼5000 events/second) and the abort rate kept below 5% for the duration of the experiment.

### 4.8 Sequences and modifications of RNA oligonucleotides

ImmRNA (5′‐pppGGAUUUCCACCUUCGGGGGAAAUCC‐3′) and 5′ triphosphate 125b‐ASO (3p‐125b‐ASO) (5′‐pppGGAAGUUAGGGUCUCAGGCCCUAACUUCC‐3′) were synthesized by in vitro transcription, as described in the next section. Anti‐miR‐125b ASO (125b‐ASO) (5′‐UCACAAGUUAGGGUCUCAGGGA‐3′), negative control RNA (NC RNA) (5′‐CAGUACUUUUGUGUAGUACAA‐3′) and FAM‐labelled NC‐ASO (5′‐FAMCAGUACUUUUGUGUAGUACAA‐3′) were synthesized with 2′ O‐methyl modification at every ribonucleotide by Shanghai GenePharma (Shanghai, China).

### 4.9 In vitro transcription (IVT) of RNA

ImmRNAs were prepared following the protocol of Yong et al. (2019). Briefly, RNAs were transcribed in vitro using T7 RNA polymerase and a pair of primers (Forward: GGATTTCCCCCGAAGGTGGAAATCCTATAGTGAGTCGTATTAC; Reverse: GTAATACGACTCACTATAGGATTTCCACCTTCGGGGGAAATCC). The reactions contain 40 mm Tris‐HCl pH 8.0, 30 mm MgCl_2_, 2 mM spermidine, 10 mM DTT, 0.01% Triton‐X100, 5 mM GTP, and 4 mM NTP (CTP, ATP and UTP), 1 μM annealed DNA template, 400–600 nM T7 RNA polymerase, and 0.2 U/ml thermostable inorganic pyrophosphatase, which react overnight at 37°C. The phenol:chloroform:isoamyl alcohol (25:24:1) was used to stop IVT reactions and extract RNAs. The RNAs were precipitated overnight at ‐80°C with three volumes of 95% ethanol in presence of 0.1% (v/v) of sodium acetate. The target RNAs were further isolated by a Hi‐TrapQ HP column and the expected bands of RNAs were excised from a 20% denaturing urea‐PAGE. Similar to immRNAs, 3p‐125b‐ASOs were prepared as described above using a dsDNA template (Sense: 5′‐GTAATACGACTCACTATAGGAAGTTAGGGTCTCAGGCT‐3′; Anti‐sense: 3′‐CATTATGCTGAGTGATATCCTTCAATCCCAGAGTCCGA‐5′). The quality of immRNAs and 3p‐125b‐ASOs were determined again on a 20% denaturing urea‐PAGE and by IFNs activity assay prior to the subsequent experiments.

To confirm the sequence of 3p‐125b‐ASO, the ASO was incubated with RNA 5′ pyrophosphohydrolase (RppH) (New England Biolabs) for 1–2 h at 37°C. The RNA was analysed using BioAnalyzer 2100 (Agilent, USA) and converted to cDNA using a NEBNext small RNA library prep kit (New England Biolabs) according to the manufacturer's instructions. The library was sequenced using Illumina HiSeq 2500 system. Sequencing reads were processed using Geneious Prime to trim the 3′ adapter sequence (TGGAATTCTCGGGTGCCAAGG). The sequences were aligned to miR‐125b ASO using Bowtie.

### 4.10 RNA loading into RBCEVs

Fifty micrograms of RBCEVs were transfected with 1 μg of RNA using REG1 transfection reagent (Carmine Therapeutics, USA) or an Exo‐Fect™ Exosome Transfection Kit (System Biosciences, Canada) according to the manufacturer's protocols. Afterwards, free RNA and transfection reagents were washed away using centrifugation at 21,000*g* for 30 min. For electroporation, 50 μg of RBCEVs were electroporated with 1 μg of RNA at 250V using a GenePulser Xcell electroporator (Bio‐Rad) with exponential program at a fixed capacitance of 100 μF with 0.4 cm cuvettes.

### 4.11 Quantification of loaded RNA in RBCEVs

Following loading with RNA, RBCEVs were incubated with 1% Triton‐X (Sigma) for 5 min at room temperature and then with heparin sulfate at a final concentration of 20 mg/ml for 1 h at 37°C. After incubation, the mixture was loaded onto a 2% Tris‐acetate‐EDTA agarose gel with GelRed^®^ nucleic acid gel stain (Sigma), separated at 100 V for 60 min and visualized with a ChemiDoc™ gel documentation system. The band fluorescence intensity was quantified using ImageJ v1.8.0.

### 4.12 Conjugation of RBCEVs with anti‐human EGFR nanobody

We have previously developed an RBCEV surface functionalization method using OaAEP1 ligase to ligate peptides with ligase‐binding motif (NGL) at the C‐terminus onto RBCEV surface (Pham et al., 2021). For OaAEP1‐mediated peptide ligation, RBCEVs were incubated for 3 h in a solution with final concentration of 2 μM ligase and 500 μl biotinylated linker peptide (Biotin‐TRNGL, GL Biochem, China) at 25°C. Following incubation, RBCEVs were washed three times with PBS using centrifugation at 21,000*g* for 20 min. The biotin‐TRNGL‐ligated RBCEVs were then incubated with streptavidin (SA) (Abcam, UK) at a final concentration of 0.1 mg/ml for 2 h at room temperature. The SA‐biotin‐RBCEVs were subsequently washed as described previously to remove free unbound streptavidin. For further functionalization, the sequences of anti‐human EGFR nanobody (α‐EGFR‐VHH) and anti‐mCherry nanobody (α‐mCherry‐VHH) were cloned with additional epitope tags for detection and purification. The VHH‐coding DNA was synthesized and inserted into pET32(a+) plasmid, following a T7 promoter, by Guangzhou IGE Biotechnology Ltd (China). Nanobodies were expressed and purified as described previously (Pham et al., 2021). After purification, α‐EGFR‐VHH or α‐mCherry‐VHH was biotinylated using a Type B‐Lightning‐Link^®^ Biotinylation Kit (Abcam) following the manufacturer's instructions. The biotin‐hEGFR‐VHH or biotin‐mCherry‐VHH was then incubated with SA‐biotin‐RBCEVs for 6 h at 4°C and washed twice with PBS. After washing, uncoated or VHH‐coated RBCEVs were incubated with 20 μM CFSE (Life Technologies) for 1 h at 37°C and washed thrice with PBS. After washing labelled RBCEVs were loaded into a size exclusion chromatographic (SEC) column (Izon, New Zealand) and eluted with PBS to wash away the unbound dye. RBCEVs, collected from SEC fraction 7–9, were washed twice with PBS by centrifugation at 21,000*g* for 15 min at 4°C. For in vivo treatment, uncoated or VHH‐coated RBCEVs were loaded with immRNA using REG1 as described earlier.

### 4.13 In vitro treatment of cancer cells with RBCEVs

A total of 50,000 4T1, CA1a or H358 cells were seeded in a 24‐well plate prior to the incubation with 0.1 μg/μl immRNA‐, 3p‐125b‐ASO‐, 125b‐ASO‐, or NC‐RNA‐loaded RBCEVs for 24–72 h in a humidified incubator at 37°C with 5% CO_2_. Cells were then harvested for immunofluorescent imaging, RT‐qPCR, or flow cytometry analysis. For dose response assay, 50,000 CA1a cells were incubated with 0.2, 0.1, 0.05, 0.025, and 0.0125 μg/μl of 125b‐ASO‐loaded RBCEVs for 24 h and then collected for RT‐qPCR analysis. For EV targeted delivery assay, 100,000 4T1‐hEGFR cells were incubated with 0.02 μg/μl uncoated or VHH‐coated CFSE‐labelled or FAM‐NC‐ASO‐loaded RBCEVs for 2 h and then collected for flow cytometry analysis. 100,000 4T1‐hEGFR cells were incubated with 0.1 μg/μl uncoated or VHH‐coated immRNA‐RBCEVs for 24 h and then collected for RT‐qPCR analysis.

### 4.14 Cell viability assay

A total of 10,000 CA1a cells were seeded in a 96‐well plate prior to incubation with 0.05 μg/μl NC‐ASO‐loaded RBCEVs. After 24, 48, and 72 h, the cells were incubated with 10% (v/v) of CCK‐8 reagent (Biosharp, China) for 2 h at 37°C with 5% CO_2_. The absorbance was measured at 450 nm using a microplate reader (Tecan, Switzerland).

### 4.15 Immunofluorescent imaging

CA1a cells were pre‐seeded on poly‐D‐lysine (Gibco, USA) coated 12 mm coverslips (Citoglass, China) 24 h prior to treatment. Following treatment with FAM‐NC‐ASO‐loaded RBCEVs, the coverslips were rinsed with fresh media, and stained with CellMask™ Deep Red Plasma Membrane Stains (Thermo Fisher Scientific) for 10 min at 37°C. Cell were rinsed twice in PBS and stained with Hoechst 33342 (Abcam) for 5 min at room temperature. The coverslips were rinsed three times with PBS before being fixed for 12 min using 4% paraformaldehyde (Alfa Aesar, USA) in PBS. The coverslips were subsequently washed three times with PBS followed by a final wash with distilled water before being mounted on slides using anti‐fade fluorescence mounting medium (Abcam). Images were acquired using an Olympus FV3000 confocal microscope (Olympus, Japan). Image acquisition was conducted using FluoView software while further analysis and quantification was conducted using ImageJ v1.8.0. Cell areas were selected as regions of interest (ROIs) based on the dilated mask of Hoechst signals. FAM signals were measured as mean pixel intensity of ROIs. Total measurement area covered 1200 to 1600 cells in each condition.

### 4.16 IFN reporter assay

A549‐Dual™ and A549‐Dual™ RIG‐I^−/−^ cells were seeded in a 96‐well plate at a density of 10,000 cells per well in culture media for 18–24 h before incubation with RBCEVs. 0.05 μg/μl immRNA‐, 3p‐125b‐ASO‐, or NC‐RNA‐loaded RBCEVs were incubated with the cells for 24 and 48 h. Lucia luciferase in the supernatant was detected by the QUANTI‐Luc reagent (InvivoGen) on a Synergy H1 microplate reader (Biotek, USA).

### 4.17 In vivo generation of cancer models and treatment with RBCEVs

All mouse experiments were performed according to experimental protocols approved by the Institutional Animal Care and Use Committee of National University of Singapore. Mice of similar ages were tagged and grouped randomly for control and test treatments. Experiments were performed in a blinded manner. Exclusion was applied to the mice that accidentally died due to anaesthesia. BALB/c mice were purchased from InVivos Pte Ltd, Singapore. NSG‐SGM3 mice (NOD.CgPrkdc < scid > Il2rg < tm1Wjl > / Tg(CMV‐IL3,CSF2,KITLG)1Eav/MloySzJ) and BALB/c nude mice (strain NU/JInv) were purchased from the Jackson Laboratory, USA.

#### 4.17.1 Intratumoral administration of RBCEVs

Female 7‐week‐old BALB/c mice were injected with 1.25 × 10^5^ 4T1 cells in the fourth MFP. After 10 days, mice were grouped randomly and injected with 2.5 mg/kg immRNA‐loaded RBCEVs, 5 mg/kg 3p‐125b‐ASO‐ loaded RBCEVs , or same amount of NC‐RNA‐loaded RBCEVs as controls intratumorally. Intratumoral injection was repeated every three days, five times in total. For PD‐L1 blockade treatment, the mice bearing 4T1 tumours were injected intraperitoneally with 2 mg/kg of anti‐mouse PD‐L1 monoclonal antibody (BioXCell, USA) one day after intratumoral injection of immRNA ‐ loaded RBCEVs. The treatment was repeated five times at intervals of three days. Tumour size was measured every two days using digital calipers. Mice were sacrificed when untreated tumours approached ∼15 mm in diameter. Tumours were collected for RNA extraction, flow cytometry analysis or imaging.

Female 7‐week‐old NSG‐SGM3 mice or nude mice were injected with 1 × 10^6^ CA1a cells or 5 × 10^6^ MDA‐MB‐468 cells, respectively, in the fourth MFP. After 14 days, mice were grouped randomly and injected with 2.5 mg/kg immRNA‐ or NC‐RNA‐loaded RBCEVs intratumorally. Intratumoral injection was repeated every three days, five times in total. Tumour size was measured every two days using digital calipers. Mice were sacrificed when untreated tumours approached ∼15 mm in diameter. Tumours were collected for RNA extraction, flow cytometry analysis and imaging.

#### 4.17.2 Intrapulmonary targeted delivery of RBCEVs

To generate a breast cancer metastasis model, 4T1 cells were transduced with a lentiviral vector (pHAGE‐EGFR, Addgene, USA), selected with puromycin (Santa Cruz) and sorted using Aria II sorter (BD Biosciences) to create a stable cell line with high expression level of human EGFR. A total of 2.5 × 10^5^ 4T1‐hEGFR cells were injected intravenously in female 7‐week‐old BALB/c mice. One day post inoculation, intrapulmonary delivery of 25 mg/kg uncoated or VHH‐coated immRNA‐RBCEVs was conducted every two days in the respective mice using a mouse Microspray Aerosolizer (Yuyan, China). After five treatments, the mice were sacrificed and the lungs were excised for RNA extraction, flow cytometry analysis or imaging. For intrapulmonary biodistribution of RBCEVs, 5 × 10^5^ 4T1‐hEGFR cells were injected intravenously in female 7‐week‐old BALB/c mice. After seven days, intrapulmonary delivery of 25 mg/kg uncoated or VHH‐coated CFSE‐RBCEVs was carried out. After 24 h, the mice were sacrificed and the lungs were excised for flow cytometry analysis.

### 4.18 Flow cytometry analysis

#### 4.18.1 RBCEV analysis

To quantify FAM‐NC‐ASO loading efficiency, 50 μg of RBCEVs were incubated with 2.5 μg of latex beads (Thermo Fisher Scientific) overnight at 4°C on a shaker, washed three times with PBS and resuspended in 100 μl of FACS buffer. Flow cytometry analysis of latex beads was performed using a CytoFlex LX cytometer (Beckman Coulter). Flow cytometric plots were generated using FlowJo v10.0.7.

#### 4.18.2 Apoptosis analysis

Following treatment with RBCEVs, cells were collected and washed with PBS. Apoptosis was determined by Annexin V (ANXV)/propidium iodide (PI) staining with the apoptosis detection kit (Life Technologies). Briefly, 50,000 treated cells were incubated with ANXV and PI in binding buffer for 15 min at 4°C. The cells were then analyzed by FACS LSRII cytometer (BD BioSciences, USA) or CytoFlexS cytometer (Beckman Coulter). Flow cytometric plots were generated using FlowJo v10.0.7.

#### 4.18.3 Immune cell analysis

Tumours were excised, washed with PBS and dissociated in DMEM media containing 10% FBS and 5 mg/ml collagenase IV (Thermo Fisher Scientific) using the GentleMACS dissociator (Miltenyi Biotec, Germany). Cells were filtered through a 70 μm strainer, blocked with anti‐mouse CD16/CD32 antibody (BioLegend), and stained with anti‐mouse CD45‐PE‐Cy7 (BioLegend), anti‐mouse CD11b‐FITC (BioLegend), anti‐mouse F4/80‐APC (BioLegend), anti‐mouse Ly6G/C‐APC (BioLegend), anti‐mouse CD3ε‐APC (BioLegend), anti‐mouse CD11c‐PB (BioLegend), anti‐mouse CD49b‐APC (BioLegend), anti‐mouse SiglecF‐PE (BioLegend), anti‐mouse CD103‐APC (BioLegend), anti‐mouse CD4‐BV421 (BioLegend), anti‐mouse CD8‐APC (BioLegend), anti‐mouse MHCII‐PE‐Cy7 (BioLegend), anti‐mouse CD206‐APC (BioLegend), anti‐mouse CD86‐APC‐Cy7 (BioLegend), anti‐FLAG‐APC antibody (BioLegend), or anti‐human EGFR nanobody for 30 min at 4°C. Cells were subsequently washed three times in FACS buffer. Cells were analyzed using FACS LSRII cytometer (BD BioSciences) or CytoFLEX S cytometer (Beckman Coulter) or CytoFLEX LX cytometer (Beckman Coulter). Dead cells were identified by staining with SYTOX™ dye (Thermo Fisher Scientific) and removed from the analysis. FCS files were analyzed using FlowJo v10.0.7, Kaluza (Beckman Coulter) or Cytobank viSNE (Beckman Coulter). Briefly, cells were first gated using an FSC‐A versus SSC‐A plot to exclude debris and dead cells. Single cells were subsequently gated via an FSC‐W versus FSC‐H plot, excluding doublets and aggregated cells. The fluorescent‐positive population of cells was subsequently gated by targeted fluorescent channels and then subjected to viSNE analysis. Equal event sampling was selected. viSNE plots for each individual parameter were downloaded from Cytobank. Cellular phenotypes were assigned to the viSNE plot based on distribution and expression characteristics using phenotypic markers.

#### 4.18.4 Ex vivo analysis of tumour‐specific T cell activity

For detection of CD69 and granzyme B expression of tumour‐infiltrated CD8^+^ T cells, CD8^+^ T cells from dissociated tumour cells were selected using CD8^+^ tumour‐infiltrating lymphocytes isolation kit (Miltenyi Biotec). The isolated CD8^+^ T cells were blocked with anti‐mouse CD16/CD32 antibody and stained with anti‐mouse CD69‐PE (BioLegend) or anti‐mouse granzyme B‐FITC antibody (BioLegend). Data were obtained using a FACS LSRII cytometer and analyzed using FlowJo.

For detection of tumour‐specific T cell activity, isolated CD8^+^ T cells were incubated with 4T1‐hEGFR‐luciferase cells at a ratio of 50:1 for 48 h. The supernatant devoid of floating cells was harvested. The quantity of IFNγ of each supernatant was analyzed using a mouse IFNγ ELISA kit (BioLegend) according to the manufacturer's instruction. The tumour cell viability was assessed using a luciferase assay kit (Promega, USA) according to the manufacturer's instruction. Data were obtained using a microplate reader (Tecan).

### 4.19 Mouse cytokine immunoassay

Mouse blood was harvested by cardiac puncture at the endpoint of in vivo treatments. The whole blood was clotted for 30 min at 37°C and the sera were isolated following centrifugation at 5,000 rpm for 20 min. The isolated sera were collected and stored at ‐80°C. The concentration of IFNβ in the sera was measured using a Recombinant Mouse IFNβ ELISA kit (BioLegend) according to the manufacturer's instruction. For multiplex immunoassay, cell culture supernatant was collected following centrifugation at 300*g* for 5 min. Tumours were excised and homogenized in cold RIPA buffer supplemented with protease inhibitor. Tumour lysates were collected using centrifugation at 12,000*g* for 20 min. The concentrations of cytokines in the collected supernatant or tumour lysates were measured using a ProcartaPlex™ Multiplex Immunoassay kit (Invitrogen, USA) according to the manufacturer's instruction. The cytokines assayed include IFNα, IFNβ, IL‐6, IL‐10, IL‐12p40, and TNFα.

### 4.20 RNA extraction and RT‐qPCR

Total RNA was extracted from cells or tissues using TRIzol (Thermo Fisher Scientific) according to the manufacturer's manuals. RNA was converted to cDNA using a high capacity cDNA reverse transcription kit (Thermo Fisher Scientific) following the manufacturer's protocol. mRNA levels were quantified using Ssofast^®^ Green qPCR kit (Bio‐Rad), normalized to *GAPDH* (for primer sequences, see Supplementary Table S1). miRNA levels were quantified using Taqman^®^ miRNA qPCR kit (Thermo Fisher Scientific), normalized to snoRNA234 (mouse) or U6B snRNA (human). All qPCR reactions were performed using a CFX96 Touch™ Real‐Time PCR Detection System (Bio‐Rad) or a QuantStudio 6 Flex Real‐Time PCR System (Life Technologies).

### 4.21 H & E staining and TUNEL assay

Following overnight fixation in 10% neutral buffered formalin (Sigma), tumour or lung tissues were sequentially dehydrated in 70%, 80%, 90%, and 100% ethanol at 37°C using a Leica TP1020 tissue processor (Leica, Germany). Samples were cleared in three baths of Histo‐Clear (National Diagnostics, USA), each for 1.5 h at 37°C, and impregnated in three baths of paraffin wax (Thermo Fisher Scientific), each for 1 h at 62°C, respectively. The paraffin blocks were sectioned at 5 μm using a Leica RM2255 rotary microtome. Sections were dried at 37°C and serially dewaxed in three baths of Histo‐Clear, then immersed in two baths of absolute ethanol and one bath of 70% ethanol, each for 10 min. Sections were rehydrated in 90%, 75%, and 50% ethanol, each for 5 min, and distilled water for 10 min. Subsequently, sections were stained with Hematoxylin (Abcam) for 5 min. After washing with water, the sections were treated with 0.3% acid alcohol, washed and blued with bluing reagent (Abcam). Sections were subsequently stained with 0.5% Eosin (Abcam) for 1 min. After washing with water, sections were dehydrated in absolute ethanol, cleared in Histo‐Clear then mounted using Histomount mounting solution (National Diagnostics). Images were acquired with a TissueFAXS PLUS slide scanner (TissueGnostics, Austria). Image acquisition was conducted using TissueFAXS viewer software while further analysis and quantification was conducted using ImageJ v1.8.0.

Apoptosis was evaluated using a TUNEL assay BrdU‐Red kit (Abcam) in combination with immunofluorescent staining. 4T1 tumour sections were washed twice with PBS. Retrieval of antigen was conducted by superheating the sections in a microwave oven for 15 min until boiling of antigen retrieval solution (Tris‐EDTA, pH 9.0) was attained. Sections were allowed to cool down in the retrieval solution for 30 min. Blocking buffer (5% normal donkey serum and 0.3% TritonX‐100 in PBS) was applied for 1 h at room temperature. After incubation with rabbit anti‐mouse α‐smooth muscle actin antibody (Abcam, dilution 1:250) overnight at 4°C, the sections were incubated with donkey anti‐rabbit secondary antibody conjugated with Alexa Fluor^®^ 647 fluorophore (Jackson ImmunoResearch, dilution 1:200) for 1 h at room temperature. Sections were then stained using the TUNEL assay kit according to the manufacturer's protocol. After staining, images were acquired with an LSM‐710 NLO confocal microscope (Zeiss, Germany) or an Olympus FV3000 confocal microscope (Olympus). Image acquisition was conducted using Zeiss Zen software or FluoView software while further analysis and quantification was conducted using ImageJ v1.8.0. Nuclei areas were selected as regions of interest (ROI) based on Hoechst signal. BrdU‐Red signals were measured as mean pixel intensity in selected ROIs.

### 4.22 Statistical analysis

All statistical analysis was performed using Student's two‐tailed *t*‐tests in GraphPad Prism 8 (GraphPad Software, CA) to determine significant differences between treated samples and control. *P*‐values less than 0.05 were considered significant, based on at least three independent replicates. In all the graphs, data are presented as median or mean and standard error of the mean (SEM). Animal experiments were repeated in groups of five to six mice. The minimum sample size of three was determined using G*Power analysis which compares the mean difference of two independent groups with α error prob = 0.05, effect size d = 5, and power = 0.95.

## CONFLICT OF INTEREST

Minh TN Le is a scientific co‐founder, advisor and shareholder of Carmine Therapeutics, a company that develops extracellular‐vesicle‐based therapies. Other authors declare no conflict of interest.

## GEOLOCATION INFORMATION

This study was done in Singapore.

## Supporting information

Supporting information.
