# Supplementary material for: Robust delivery of RIG‐I agonists using extracellular vesicles for anti‐cancer immunotherapy
Source: J Extracell Vesicles. 2022 Apr 16;11(4):e12187. doi: 10.1002/jev2.12187 (PMC9013404; doi:10.1002/jev2.12187)
Supplement: Supplementary file 1 — Supporting information. [file JEV2-11-e12187-s001.pdf]

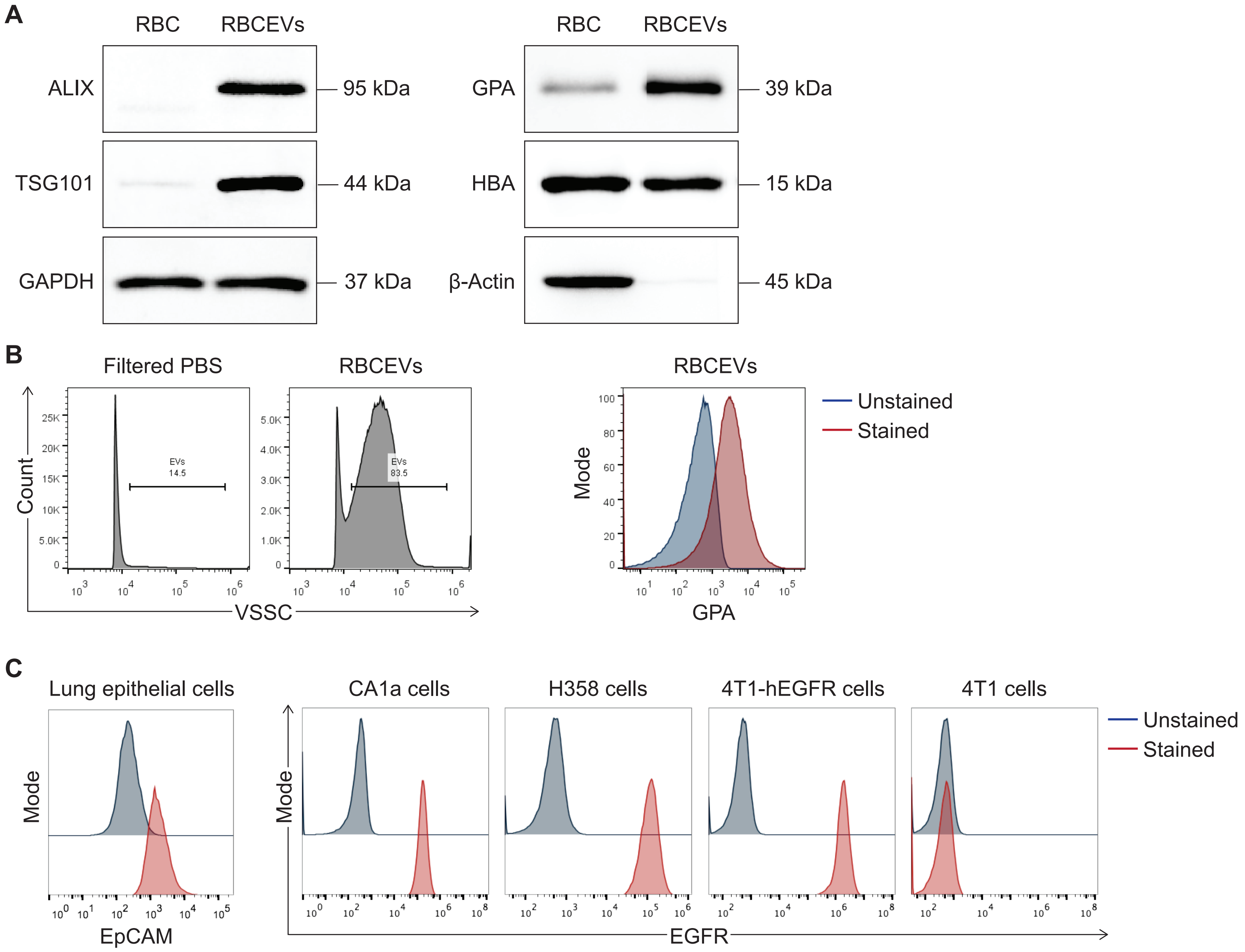

Figure S1| Characterization of RBCEVs, mouse lung epithelial cells and α-EGFR-VHH binding affinity
(A) Western blot of ALIX, TSG101, GPA, HBA, β-actin and GAPDH in RBCs and RBCEVs. **(B)** Single-EV flow cytometry analysis of GPA in RBCEVs with a gating strategy to obtain a distinct population of RBCEVs from the background noise. **(C)** Flow cytometry analysis of EpCAM expression in mouse lung epithelial cells and α-EGFR-VHH binding affinity to multiple cells.

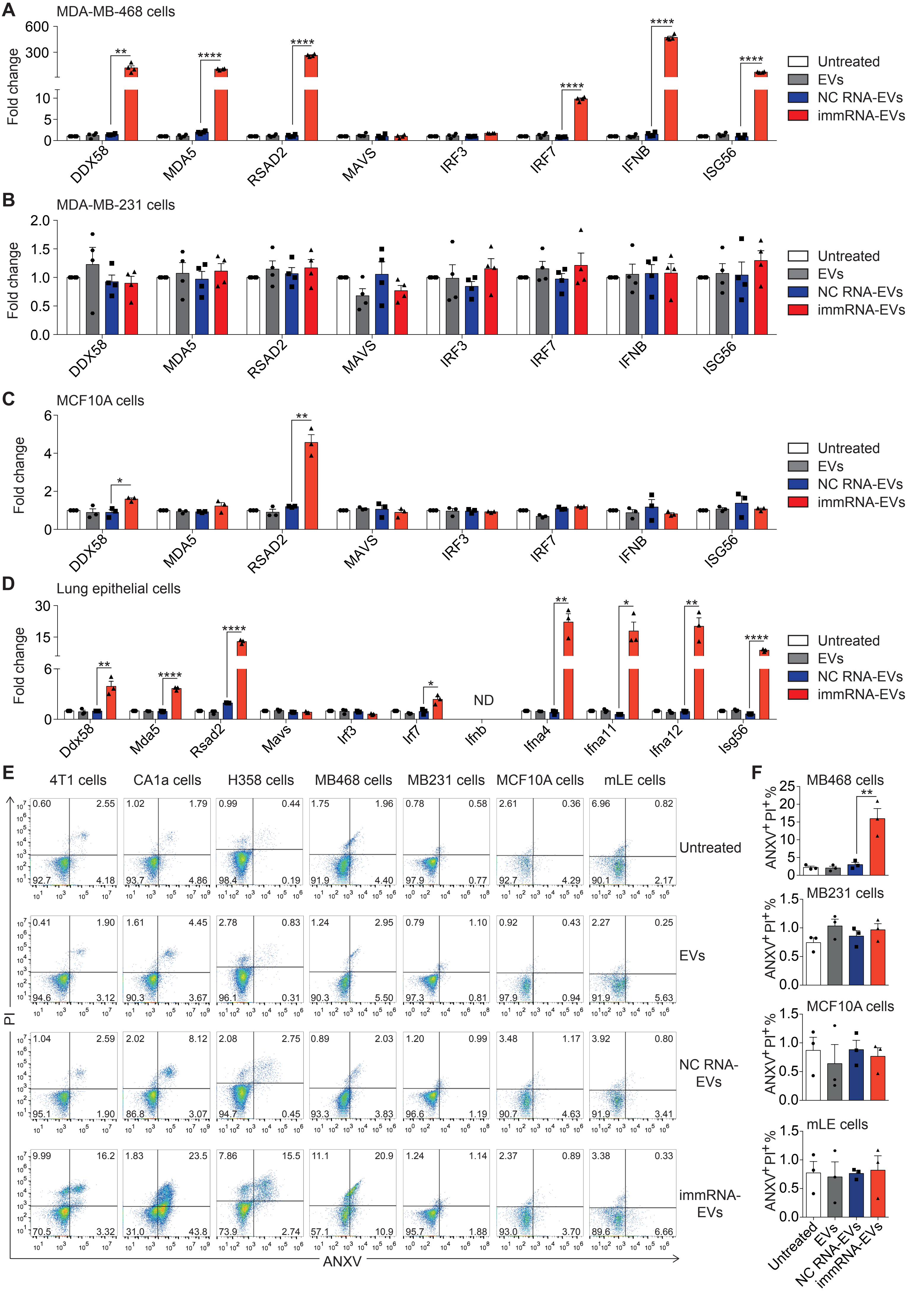
A
MDA-MB-468 cells
Fold change
DDX58
MDA5
RSAD2
MAVS
IRF3
IRF7
IFNB
ISG56
Untreated
EVs
NC RNA-EVs
immRNA-EVs
B
MDA-MB-231 cells
C
MCF10A cells
D
Lung epithelial cells
Ddx58
Mda5
Rsad2
Mavs
Irf3
Irf7
Ifnb
Ifna4
Ifna11
Ifna12
Isg56
ND
E
4T1 cells
CA1a cells
H358 cells
MB468 cells
MB231 cells
MCF10A cells
mLE cells
PI
ANXV
Untreated
0.60 2.55 92.7 4.18
1.02 1.79 93.7 4.86
0.99 0.44 98.4 0.19
1.75 1.96 91.9 4.40
0.78 0.58 97.9 0.77
2.61 0.36 92.7 4.29
6.96 0.82 90.1 2.17
EVs
0.41 1.90 94.6 3.12
1.61 4.45 90.3 3.67
2.78 0.83 96.1 0.31
1.24 2.95 90.3 5.50
0.79 1.10 97.3 0.81
0.92 0.43 97.9 0.94
2.27 0.25 91.9 5.63
NC RNA-EVs
1.04 2.59 95.1 1.90
2.02 8.12 86.8 3.07
2.08 2.75 94.7 0.45
0.89 2.03 93.3 3.83
1.20 0.99 96.6 1.19
3.48 1.17 90.7 4.63
3.92 0.80 91.9 3.41
immRNA-EVs
9.99 16.2 70.5 3.32
1.83 23.5 31.0 43.8
7.86 15.5 73.9 2.74
11.1 20.9 57.1 10.9
1.24 1.14 95.7 1.88
2.37 0.89 93.0 3.70
3.38 0.33 89.6 6.66
F
MB468 cells
MB231 cells
MCF10A cells
mLE cells
ANXV+PI+ %
Untreated
EVs
NC RNA-EVs
immRNA-EVs

Figure S2| The effects of immRNA-loaded RBCEVs on cancer cells and non-malignant cells

(A-D) qPCR analysis of *DDX58* and its downstream effectors relative to *GAPDH* in MDA-MB-468 (A), MDA-MB-231 (B), MCF10A (C) and mouse lung epithelial cells (D) treated with 0.1 µg/µL unloaded or NC-RNA-loaded, immRNA-loaded RBCEVs for 24 hours (n = 3-4, RNA loaded using REG1) ND, not detected. **(E)** Representative flow cytometric plots of ANXV/PI staining in 4T1, CA1a, H358, MDA-MB-468 (MB468), MDA-MB-231 (MB231), MCF10A and mouse lung epithelial (mLE) cells treated with 0.1 µg/µL unloaded RBCEVs, NC RNA-loaded RBCEVs and immRNA-loaded RBCEVs for 72 hours. **(F)** Average percentage of $ANXV^{+}PI^{+}$ population in MB468, MB231, MCF10A and mLE cells treated with RBCEVs as in (E) (n = 3). All bar graphs represent mean ± SEM. **P* < 0.05, ***P* < 0.01 and *****P* < 0.0001 determined by Student's two-tailed *t*-test.

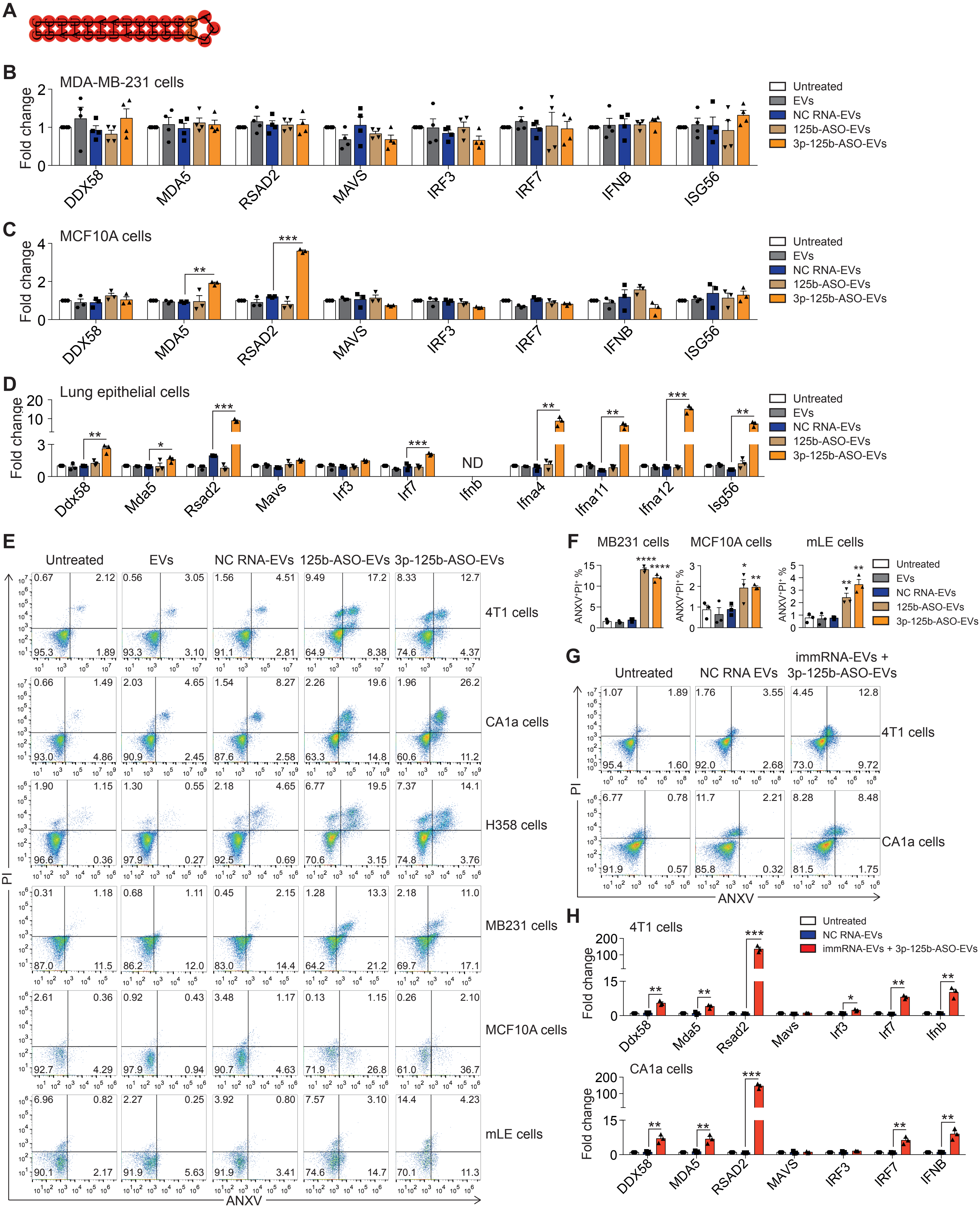
A
B
MDA-MB-231 cells
Fold change
DDX58
MDA5
RSAD2
MAVS
IRF3
IRF7
IFNB
ISG56
Untreated
EVs
NC RNA-EVs
125b-ASO-EVs
3p-125b-ASO-EVs
C
MCF10A cells
Fold change
DDX58
MDA5
RSAD2
MAVS
IRF3
IRF7
IFNB
ISG56
D
Lung epithelial cells
Fold change
Ddx58
Mda5
Rsad2
Mavs
Irf3
Irf7
Ifnb
ND
Ifna4
Ifna11
Ifna12
Isg56
E
Untreated
EVs
NC RNA-EVs
125b-ASO-EVs
3p-125b-ASO-EVs
4T1 cells
CA1a cells
H358 cells
MB231 cells
MCF10A cells
mLE cells
PI
ANXV
F
MB231 cells
MCF10A cells
mLE cells
ANXV⁺PI⁺ %
G
Untreated
NC RNA EVs
immRNA-EVs + 3p-125b-ASO-EVs
4T1 cells
CA1a cells
PI
ANXV
H
4T1 cells
Fold change
Ddx58
Mda5
Rsad2
Mavs
Irf3
Irf7
Ifnb
Untreated
NC RNA-EVs
immRNA-EVs + 3p-125b-ASO-EVs
CA1a cells
DDX58
MDA5
RSAD2
MAVS
IRF3
IRF7
IFNB

Figure S3| The effects of 3p-125b-ASO-loaded RBCEVs on cancer cells and non-malignant cells

(A) Secondary structure of 3p-125b-ASO. **(B-D)** qPCR analysis of *DDX58* and its downstream effectors relative to *GAPDH* in MDA-MB-231 (B), MCF10A (C) and mouse lung epithelial cells (D) treated with 0.1 µg/µL unloaded or NC-RNA-loaded, 125b-ASO-loaded and 3p-125b-ASO-loaded RBCEVs for 24 hours (n = 3-4, RNA loaded using REG1) ND, not detected. **(E)** Representative flow cytometric plots of ANXV/PI staining in 4T1, CA1a, H358, MDA-MB-231 (MB231), MCF10A and mouse lung epithelial (mLE) cells treated with 0.1 µg/µL unloaded or NC RNA-loaded, 125b-ASO-loaded and 3p-125b-ASO-loaded RBCEVs for 72 hours. **(F)** Average percentage of $ANXV^{+}PI^{+}$ population in MB231, MCF10A and mLE cells treated with RBCEVs as in (E) (n = 3). **(G)** Representative flow cytometric plots of ANXV/PI staining in 4T1 cells and CA1a cells after a treatment with combined 3p-125-ASO- and immRNA-loaded RBCEVs for 72 hours. **(H)** qPCR analysis of RIG-I pathway gene expression in 4T1 cells and CA1a cells after a treatment with combined 3p-125-ASO- and immRNA-loaded RBCEVs for 24 hours. All bar graphs represent mean ± SEM. *$P < 0.05$, **$P < 0.01$, ***$P < 0.001$ and ****$P < 0.0001$ determined by Student's two-tailed *t*-test.

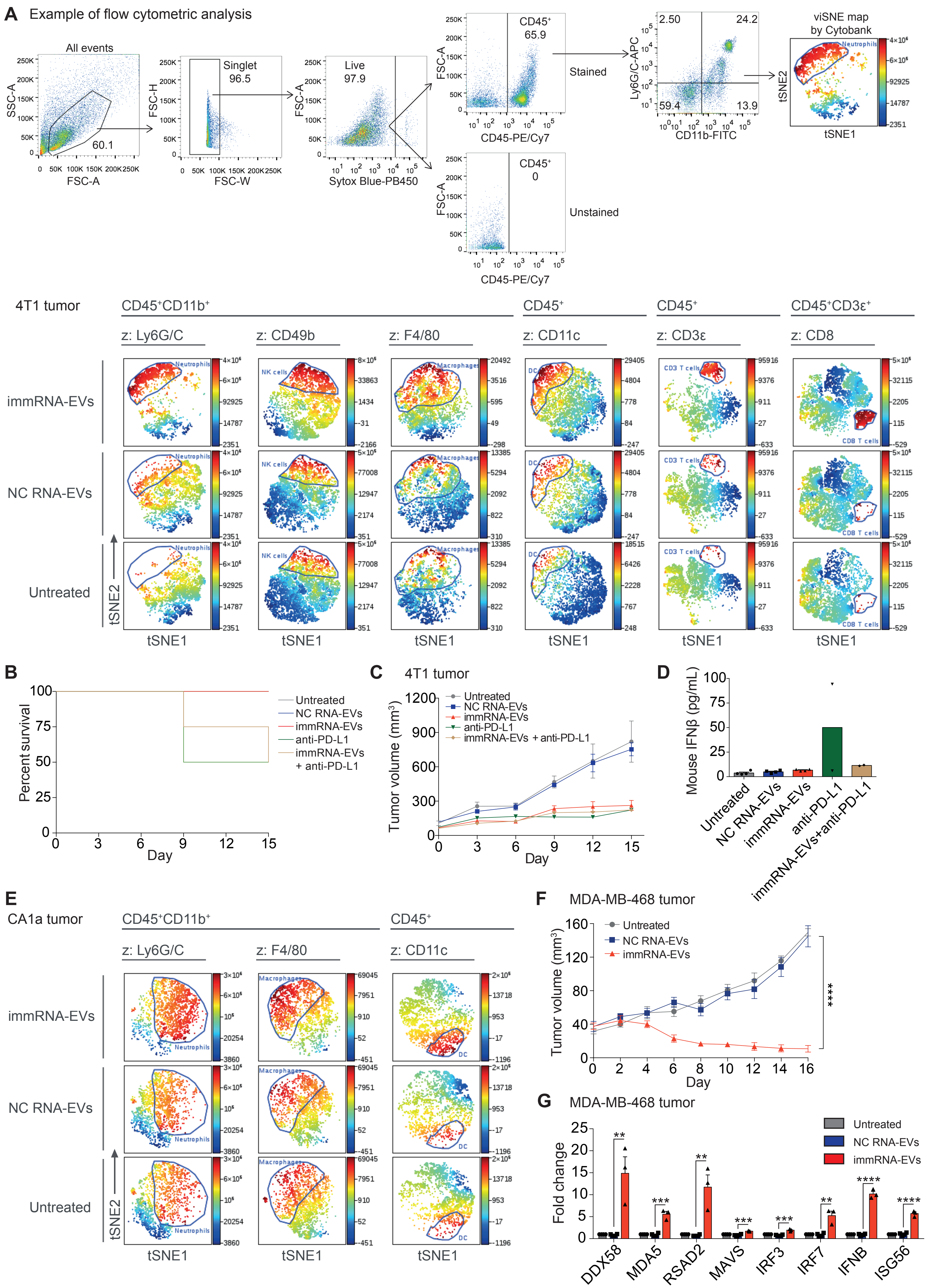
A
Example of flow cytometric analysis
All events
SSC-A
FSC-A
60.1
Singlet
96.5
FSC-H
FSC-W
Live
97.9
FSC-A
Sytox Blue-PB450
CD45+
65.9
CD45-PE/Cy7
Stained
CD45+
0
Unstained
Ly6G/C-APC
CD11b-FITC
2.50
24.2
59.4
13.9
viSNE map
by Cytobank
Neutrophils
tSNE2
tSNE1
4T1 tumor
CD45+CD11b+
CD45+
CD45+
CD45+CD3ε+
z: Ly6G/C
z: CD49b
z: F4/80
z: CD11c
z: CD3ε
z: CD8
immRNA-EVs
NC RNA-EVs
Untreated
Neutrophils
NK cells
Macrophages
DC
CD3 T cells
CD8 T cells
B
Percent survival
Day
Untreated
NC RNA-EVs
immRNA-EVs
anti-PD-L1
immRNA-EVs
+ anti-PD-L1
C
4T1 tumor
Tumor volume (mm3)
Day
Untreated
NC RNA-EVs
immRNA-EVs
anti-PD-L1
immRNA-EVs + anti-PD-L1
D
Mouse IFNβ (pg/mL)
Untreated
NC RNA-EVs
immRNA-EVs
anti-PD-L1
immRNA-EVs+anti-PD-L1
E
CA1a tumor
CD45+CD11b+
CD45+
z: Ly6G/C
z: F4/80
z: CD11c
immRNA-EVs
NC RNA-EVs
Untreated
Neutrophils
Macrophages
DC
tSNE2
tSNE1
F
MDA-MB-468 tumor
Tumor volume (mm3)
Day
Untreated
NC RNA-EVs
immRNA-EVs
****
G
MDA-MB-468 tumor
Fold change
Untreated
NC RNA-EVs
immRNA-EVs
DDX58
MDA5
RSAD2
MAVS
IRF3
IRF7
IFNB
ISG56
**
***
**
***
***
**
****
****

Figure S4| Therapeutic efficacy of immRNA-loaded RBCEVs in mammary tumors

(A) Flow cytometry analysis of immune cells in 4T1 mammary tumors treated with immRNA-loaded RBCEVs as in Figure 4. Cells were gated based on FSC-A and SSC-A to exclude the debris and dead cells. The single cells were further gated based on FSC-W and FSC-H to exclude doublets and aggregates. The live cells were gated from singlet population base on Sytox^{TM} Blue negative. Subsequently, the fluorescence of phenotypic markers for immune cells were gated based on the fluorescent channels and subjected to viSNE analysis. **(B)** Survival of mice with orthotopic 4T1 tumors treated with 2.5 mg/kg NC-RNA-RBCEVs (i.t.), 2.5 mg/kg immRNA-RBCEVs (i.t.), 2 mg/kg anti-PD-L1 antibody (i.p.) as well as 2.5 mg/kg immRNA-RBCEVs (i.t.) in combination with 2 mg/kg anti-PD-L1 antibody (i.p.) (n = 2-4 mice). **(C)** Tumor growth of mice treated as in (B) (n = 2-4 mice). **(D)** ELISA quantification of IFNβ in the sera of mice bearing 4T1 tumors treated as in (B) (n = 2-4 mice). **(E)** Representative viSNE plots of immune cells in CA1a mammary tumors treated with immRNA-loaded RBCEVs as in Figure 4. **(F)** Volume of MDA-MB-468 tumors injected intratumorally with 2.5 mg/kg RBCEVs containing immRNA or NC RNA every three days (n = 3-4 mice). **(G)** qPCR analysis of the RIG-I pathway gene expression relative to *GAPDH* in untreated and treated MDA-MB-468 tumors (n = 3-4 mice). All bar graphs represent mean ± SEM. $^{**}P < 0.01$, $^{***}P < 0.001$ and $^{****}P < 0.0001$ determined by Student's two-tailed *t*-test.

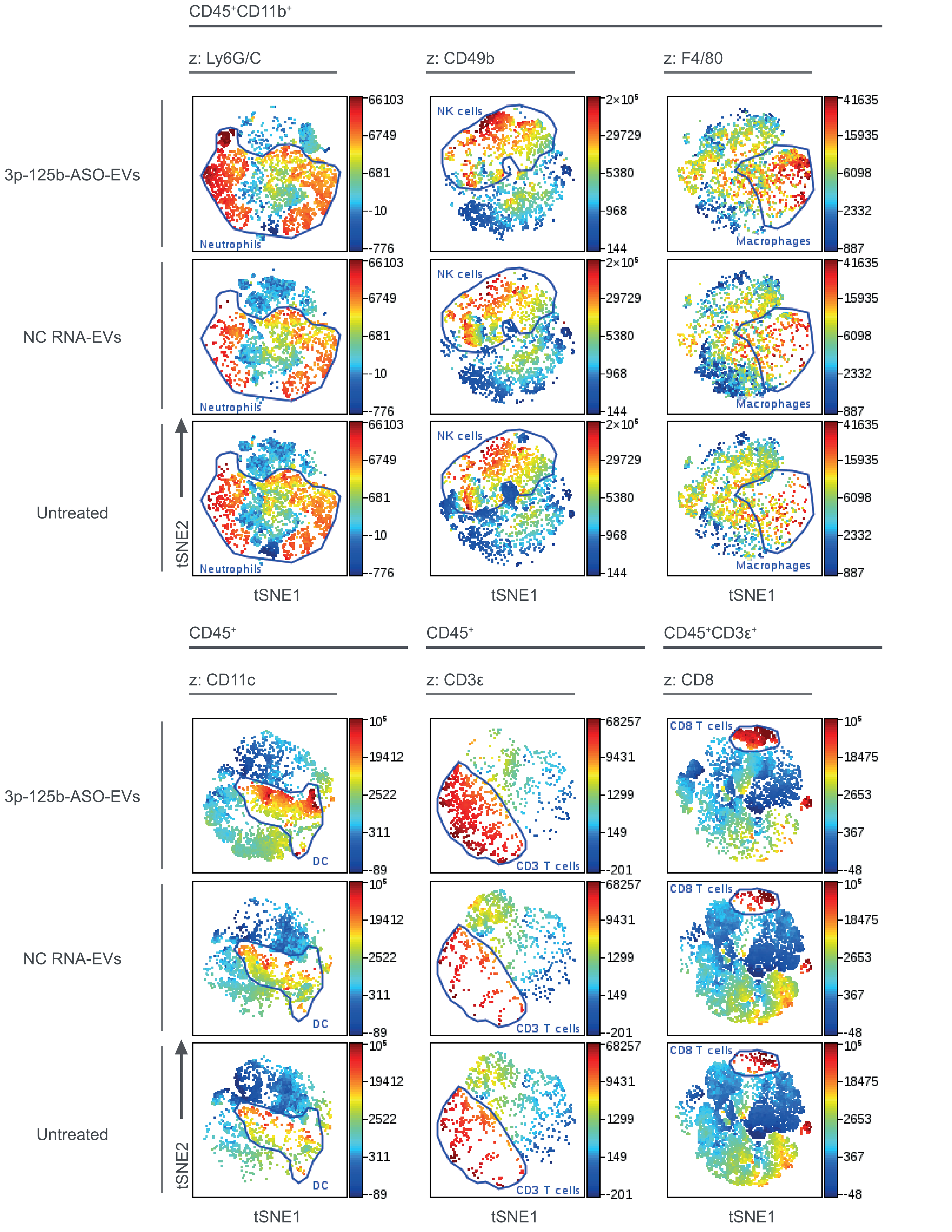

Figure S5| Flow cytometry analysis of immune cells in 4T1 mammary tumors treated with 3p-125b-ASO-loaded RBCEVs
Representative viSNE plots of immune cells in 4T1 mammary tumors treated with 3p-125b-ASO-loaded RBCEVs.

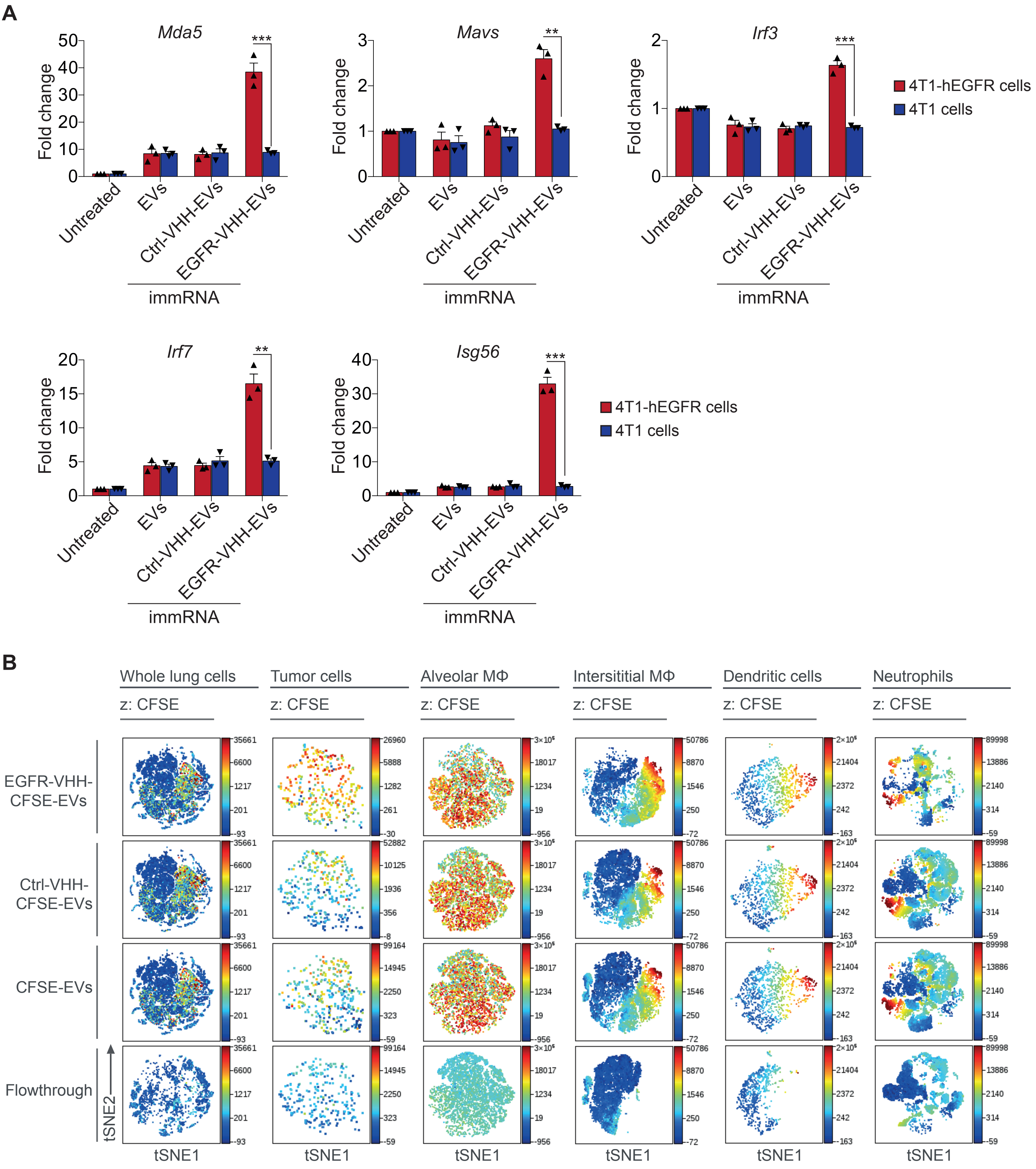

Figure S6| EGFR-VHH-RBCEVs target 4T1-hEGFR cells *in vitro* and 4T1-hEGFR lung metastatic tumors *in vivo*
(A) qPCR analysis of the RIG-I pathway gene expression relative to *Gapdh* in 4T1-hEGFR cells and parental 4T1 cells treated with 0.1 µg/µL uncoated, Ctrl-VHH-coated and EGFR-VHH-coated RBCEVs containing immRNA (n = 3). All bar graphs represent mean ± SEM. $^{**}P < 0.01$ and $^{***}P < 0.001$ determined by Student's two-tailed *t*-test. **(B)** Representative viSNE plots of biodistribution of EGFR-VHH-coated CFSE-RBCEVs in respective cell type in the lungs with 4T1-hEGFR metastatic tumors.

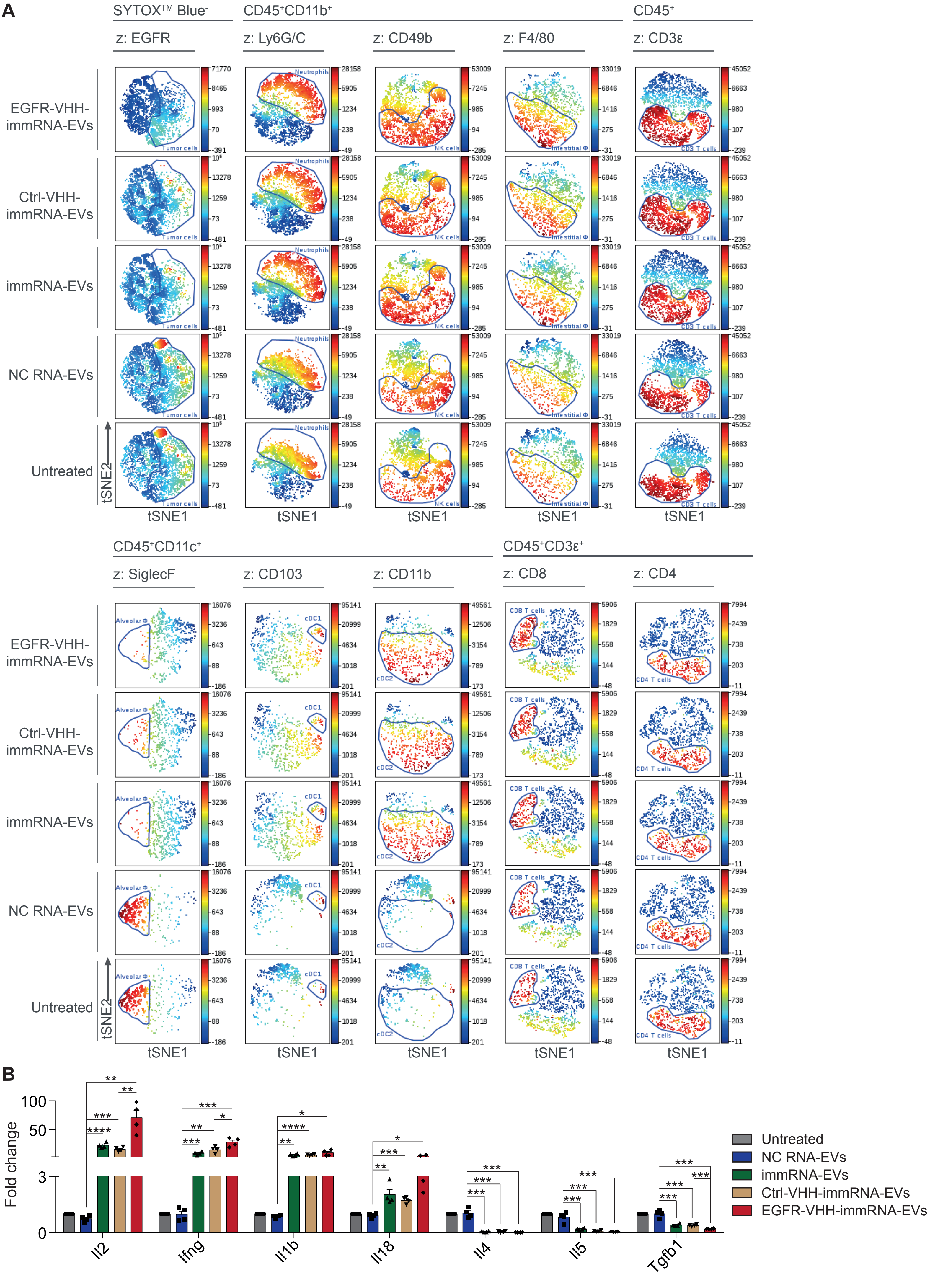
A
SYTOX™ Blue⁻
CD45⁺CD11b⁺
CD45⁺
z: EGFR
z: Ly6G/C
z: CD49b
z: F4/80
z: CD3ε
EGFR-VHH-immRNA-EVs
Ctrl-VHH-immRNA-EVs
immRNA-EVs
NC RNA-EVs
Untreated
Tumor cells
Neutrophils
NK cells
Interstitial Φ
CD3 T cells
tSNE1
tSNE2
CD45⁺CD11c⁺
CD45⁺CD3ε⁺
z: SiglecF
z: CD103
z: CD11b
z: CD8
z: CD4
Alveolar Φ
cDC1
cDC2
CD8 T cells
CD4 T cells
B
Fold change
Il2
Ifng
Il1b
Il18
Il4
Il5
Tgfb1
Untreated
NC RNA-EVs
immRNA-EVs
Ctrl-VHH-immRNA-EVs
EGFR-VHH-immRNA-EVs

Figure S7| Flow cytometry analysis of immune cells in the lungs with 4T1 metastatic tumors treated with EGFR-VHH-immRNA-RBCEVs

(A) Representative viSNE plots of respective cell type in the lungs with 4T1 metastatic tumors treated with EGFR-VHH-immRNA-RBCEVs. **(B)** qPCR analysis of cytokine gene expression relative to *Gapdh* in the lungs of mice with 4T1-hEGFR metastatic tumors (n = 4 mice). All bar graphs represent mean ± SEM. *$P < 0.05$, **$P < 0.01$, ***$P < 0.001$ and ****$P < 0.0001$ determined by Student's two-tailed *t*-test.

Table S1| List of primers

Genes	F/R	Mouse	Human
GAPDH	F	AGGTCGGTGTGAACGGATTTG	GGAGCGAGATCCCTCCAAAAT
	R	TGTAGACCATGTAGTTGAGGTCA	GGCTGTTGTCATACTTCTCATGG
DDX58	F	GAG AGT CAC GGG ACC CAC T	GCCATTACACTGTGCTTGGAGA
	R	CGG TCT TAG CAT CTC CAA CG	CCAGTTGCAATATCCTCCACCA
MDA5	F	TGATGCACTATTCCAAGAACTAAC	GAGCAACTTCTTTCAACCACAG
	R	TCTGTGAGACGAGTTAGCCAAG	CACTTCCTTCTGCCAAACTTG
RSAD2	F	ACAGCCAAGACATCCTTCGT	CACAAAGAAGTGTCCTGCTTGGT
	R	AAAAGTTGATCTTCTCCAAACCA	AAGCGCATATATTCATCCAGAAT
MAVS	F	CTGCCTCACAGCTAGTGACC	AGGAGACAGATGGAGACACA
	R	CCGGCGCTGGAGATTATTG	CAGAACTGGGCAGTACCC
IFNB	F	AACCTCACCTACAGGGCGGACTTC	CTCTCCTGTTGTGCTTCTCC
	R	TCCCACGTCAATCTTTCCTCTTGCT	GTCAAAGTTCATCCTGTCCTTG
IRF3	F	CGGAAAGAAGTGTTGCGGTT	ACCAGCCGTGGACCAAGAG
	R	TTTTCCTGGGAGTGAGGCAG	TACCAAGGCCCTGAGGCAC
IRF7	F	AGGGCGTTTTATCTTGCG	TGGTCCTGGTGAAGCTGGAA
	R	TGGAGCCCAGCATTTTCTCT	GATGTCGTCATAGAGGCTGTTGG
ISG56	F	TACAGGCTGGAGTGTGCTGAGA	TAGCCAACATGTCCTCACAGAC
	R	CTCCACTTTCAGAGCCTTCGCA	TCTTCTACCACTGGTTTCATGC
IL4	F	ATCATCGGCATTTTGAACGAGG	
	R	GCAGCTCCATGAGAACACTA	
IL5	F	CTCTGTTGACAAGCAATGAGACG	
	R	TCTTCAGTATGTCTAGCCCCTG	
IFNG	F	GCCACGGCACAGTCATTGA	
	R	TGCTGATGGCCTGATTGTCTT	
IL2	F	GTGCTCCTTGTCAACAGCG	
	R	GGGGAGTTTCAGGTTCCTGTA	
TGFB1	F	CTTCAATACGTCAGACATTCGGG	
	R	GTAACGCCAGGAATTGTTGCTA	
IL1B	F	GCAACTGTTCCTGAACTCAACT	
	R	ATCTTTTGGGGTCCGTCAACT	
IL18	F	GACTCTTGCGTCAACTTCAAGG	
	R	CAGGCTGTCTTTTGTCAACGA	
IL10	F	AGCCTTATCGGAAATGATCCAGT	
	R	GGCCTTGTAGACACCTTGGT	
IL6	F	TCTATACCACTTCACAAGTCGGA	
	R	TCTATACCACTTCACAAGTCGGA	
IL12B	F	TGGTTTGCCATCGTTTTGCTG	
	R	ACAGGTGAGGTTCACTGTTTCT	

TNF	F	CCTGTAGCCCACGTCGTAG
	R	GGGAGTAGACAAGGTACAACCC
IFNA4	F	CTGGTAATGATGAGCTACTACTG<br>G
	R	CCTTCTCCAAGGGGAATCCAA
IFNA11	F	GGTCCTGGCACAAATGAGGA
	R	TCCAAGCAGCAGATGAGTCC
IFNA12	F	AAGACTGAGTGAGAAGGAGTGAG
	R	GAGATGCCAGAATTTGAGCAGTG
